# RBBP6‐Mediated ERRα Degradation Contributes to Mitochondrial Injury in Renal Tubular Cells in Diabetic Kidney Disease

**DOI:** 10.1002/advs.202405153

**Published:** 2024-10-23

**Authors:** Hongtu Hu, Jijia Hu, Zhaowei Chen, Keju Yang, Zijing Zhu, Yiqun Hao, Zongwei Zhang, Weiwei Li, Zhuan Peng, Yun Cao, Xiaoling Sun, Fangcheng Zhang, Qingjia Chi, Guohua Ding, Wei Liang

**Affiliations:** ^1^ Division of Nephrology Renmin Hospital of Wuhan University Wuhan 430060 China; ^2^ Key Clinical Research Center of Kidney Disease Wuhan 430060 China; ^3^ Central Laboratory Renmin Hospital of Wuhan University Wuhan 430060 China; ^4^ The First College of Clinical Medical Science China Three Gorges University Yichang 443000 China; ^5^ Department of Nephrology Hainan General Hospital (Hainan Affiliated Hospital of Hainan Medical College) Haikou 100053 China; ^6^ Ultrastructural Pathology Center Renmin Hospital of Wuhan University Wuhan 430060 China; ^7^ Department of Mechanics and Engineering Structure Wuhan University of Technology Wuhan 430070 China

**Keywords:** diabetic kidney disease, estrogen‐related receptor α, mitochondrial dysfunction, proximal renal tubular cells, RBBP6, ubiquitination

## Abstract

Diabetic Kidney Disease (DKD), a major precursor to end‐stage renal disease, involves mitochondrial dysfunction in proximal renal tubular cells (PTCs), contributing to its pathogenesis. Estrogen‐related receptor α (ERRα) is essential for mitochondrial integrity in PTCs, yet its regulation in DKD is poorly understood. This study investigates ERRα expression and its regulatory mechanisms in DKD, assessing its therapeutic potential. Using genetic, biochemical, and cellular approaches, ERRα expression Was examined in human DKD specimens and DKD mouse models. We identified the E3 ubiquitin ligase retinoblastoma binding protein 6 (RBBP6) as a regulator of ERRα, promoting its degradation through K48‐linked polyubiquitination at the K100 residue. This degradation pathway significantly contributed to mitochondrial injury in PTCs of DKD models. Notably, conditional ERRα overexpression or RBBP6 inhibition markedly reduced mitochondrial damage in diabetic mice, highlighting ERRα’s protective role in maintaining mitochondrial integrity. The interaction between RBBP6 and ERRα opens new therapeutic avenues, suggesting that modulating RBBP6‐ERRα interactions could be a strategy for preserving mitochondrial function and slowing DKD progression.

## Graphical abstract

Hu et al. explore the role of estrogen‐related receptor α (ERRα) in diabetic kidney disease (DKD), revealing retinoblastoma binding protein 6 (RBBP6) as a key regulator of ERRα degradation. Targeting RBBP6–ERRα interactions shows promise in preserving mitochondrial function and mitigating DKD progression, offering potential therapeutic avenues for combating this disease.

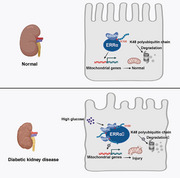

## 1 Introduction

Diabetic kidney disease (DKD) emerges as a predominant complication of diabetes, marking the leading cause of end‐stage renal disease globally.^[^ ^1^ ^]^ The etiology of DKD encompasses a complex interplay of factors, among which mitochondrial dysfunction in proximal renal tubular cells (PTCs) has gained significant attention.^[^ ^2^ ^]^ Characterized by a high mitochondrial density, PTCs play a pivotal role in energy metabolism, including fatty acid oxidation and ion transport, critical for renal function.^[^ ^3^ ^]^ The advent of sodium‐glucose cotransporter 2 (SGLT2) inhibitors, which target PTCs to attenuate mitochondrial distress, has highlighted these cells' importance in the DKD pathophysiology.^[^ ^4^ ^]^ Despite these advancements, the intricate mechanisms precipitating mitochondrial impairment in PTCs within the diabetic milieu remain to be fully elucidated.

Estrogen‐related receptor alpha (ERRα), an orphan nuclear receptor, is notably enriched in metabolically active tissues such as the kidney, liver, and skeletal muscle.^[^ ^5^ ^]^ Within the renal architecture, ERRα expression is particularly pronounced in PTCs, playing a critical role in renal physiology. Emerging studies underscore ERRα’s pivotal function in maintaining renal integrity, primarily through modulating mitochondrial structure and bioenergetics within PTCs.^[^ ^6^ ^]^ The downregulation of ERRα in these cells has been linked to a spectrum of renal insults, suggesting its protective role against cellular damage.^[^ ^6^, ^7^, ^8^, ^9^ ^]^ Despite these insights, the specific mechanisms through which ERRα expression is regulated in DKD have yet to be fully delineated.

The ubiquitin‐proteasome system (UPS) plays a critical role in the pathogenesis of various kidney diseases, including renal fibrosis, acute kidney injury, and DKD.^[^ ^10^ ^]^ Notably, an upregulation of the UPS has been observed in DKD,^[^ ^11^ ^]^ and targeting this system has shown promise in decelerating the disease's progression.^[^ ^12^ ^]^ Recent studies have shed light on the UPS's role in modulating the expression and functionality of ERRα, suggesting its involvement in renal pathology.^[^ ^13^, ^14^ ^]^ Nonetheless, the specific targets through which the UPS influences ERRα and its implications for DKD's pathophysiology remain to be clearly defined.

Our research demonstrates a significant reduction in ERRα expression across DKD patient samples and animal models, establishing a notable correlation between ERRα levels and key clinical DKD markers. We unveiled the interaction between ERRα and the E3 ubiquitin ligase RBBP6 through advanced mass spectrometry techniques, which targets ERRα for degradation via K48‐linked polyubiquitination at the K100 residue. This study is pioneering in elucidating RBBP6's role in ERRα degradation and its contribution to mitochondrial damage within DKD, marking a significant advancement in our understanding of DKD's molecular underpinnings.

## 2 Results

### 2.1 Renal Pathological Changes and ERRα Expression in DKD

**Figure 1:**
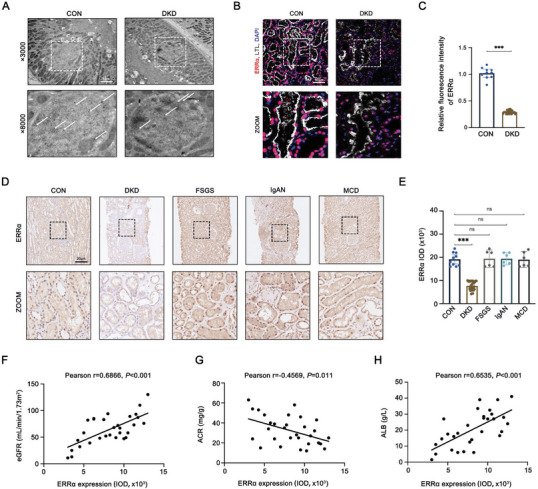
Renal pathological changes and ERRα expression in DKD. A) TEM analyses of immuno‐gold labeling of ERRα in PTCs in DKD (*n* = 30) and control (*n* = 10) group patients. B,C) Representative immunofluorescent images and quantification of LTL (gray) and ERRα (red) in PTCs from DKD (*n* = 30) and control (*n* = 10) group patients. D) Representative immunohistochemistry images of ERRα in PTCs from control (*n* = 10), DKD (*n* = 30), focal segmental glomerular sclerosis (FSGS, *n* = 6), IgA nephritis (IgAN, *n* = 6), and minimal change disease (MCD, *n* = 6) group patients. E) Immunohistochemical semi‐quantitative IOD analysis of ERRα. Correlation between F) ERRα expression and estimated glomerular filtration rate (eGFR) (*n* = 30), G) urinary creatinine‐protein ratio (ACR) (*n* = 30), and H) serum albumin (ALB) (*n* = 30) in patients with DKD. Data are presented as mean ± SEM. ns: *p* > 0.05; ***p < 0.001.

In DKD patients, we observed significant pathological changes in kidney tissues through various staining techniques, including HE, PAS, PASM, and Masson, revealing pronounced proliferation of mesangial cells, increased glycogen and collagen fiber depositions, and basement membrane thickening, when compared to control kidney tissues from nephrectomy patients (Figure S1A–F, Supporting Information). These findings underscore the pivotal role of mitochondrial injury within PTCs in DKD pathogenesis.^[^ ^15^ ^]^ Mitochondrial integrity in PTCs was further examined, revealing markedly reduced expression of the mitochondrial markers TOM20 and PGC1α, key components of mitochondrial function and biogenesis, respectively, in DKD‐affected kidneys, aligned with LTL‐positive proximal tubules (Figure S1G–I, Supporting Information). Transmission electron microscopy (TEM) analysis revealed abnormalities of mitochondrial structure and density in PTCs with DKD, including vacuolization, cristae breakage, and swelling of mitochondria, as well as a significant reduction in mitochondrial number (Figure S1J, Supporting Information). Using immuno‐gold labeling TEM, we localized ERRα expression predominantly within the nuclei of PTCs, noting a decrease in ERRα expression in DKD subjects compared to controls (**Figure** **1**A). This specific downregulation of ERRα in PTCs of DKD patients was corroborated by three‐color immunofluorescence staining, contrasting sharply with controls (Figure 1B,C). We extended our investigation to include non‐DKD renal diseases such as focal segmental glomerular sclerosis, IgA nephropathy, and minimal change disease, revealing a unique downregulation of ERRα, specifically in DKD (Figure 1D,E). Correlation analyses established significant positive associations between ERRα expression and renal function markers, such as estimated glomerular filtration rate (*r* = 0.6866, *p* < 0.001) and serum albumin (*r* = 0.6535, *p* < 0.001) and an inverse relationship with urinary albumin‐to‐creatinine ratio (ACR) (*r* = −0.4569, *p* = 0.011) (Figure 1F,H). These findings collectively highlight the critical role of ERRα in DKD, distinguishing its impact from other renal diseases and underscoring its strong association with key clinical indicators in DKD patients.

### 2.2 Downregulation of ERRα in Diabetic Kidney Disease Animal Models Linked to Mitochondrial Dysfunction in Proximal Tubular Cells

**Figure 2:**
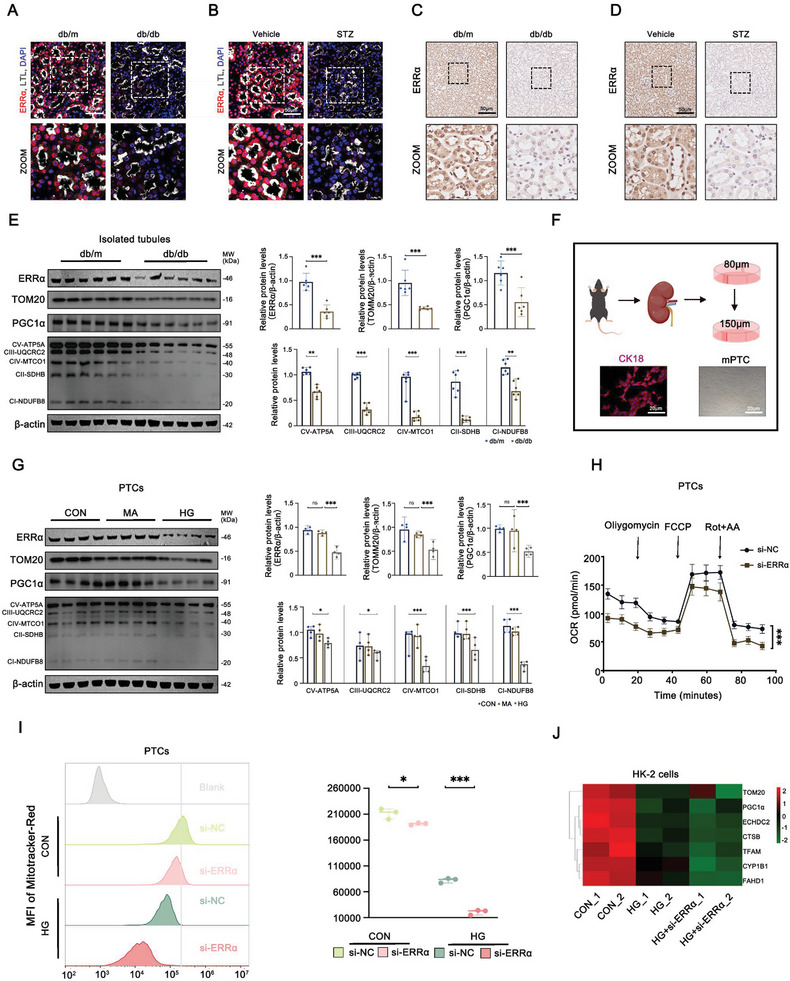
ERRα is downregulated in diabetic animal models and associated with mitochondrial function. A) Representative immunofluorescent images and quantification of LTL (gray) and ERRα (red) in PTCs from db/m (*n* = 6) and db/db (*n* = 6) mice. B) Representative immunofluorescent images and quantification of LTL (green) and ERRα (red) in PTCs from vehicle (*n* = 6) and STZ (*n* = 6) mice. C) Representative immunohistochemistry images of ERRα in PTCs from db/m and db/db mice (*n* = 6). D) Representative immunohistochemistry images of ERRα in PTCs from vehicle and STZ mice (*n* = 6). E) Representative Western blot and densitometric analysis of ERRα, TOM20, PGC1α, and OXPHOS (CI‐NDUFB8, CII‐SDHB, CIII‐UQCRC2, CIV‐MTCO1, CV‐ATP5A) in renal tubules from db/m and db/db mice (*n* = 6). F) A schematic diagram showing the isolation of primary PTCs. G) Representative Western blot and densitometric analysis of ERRα, TOM20, PGC1α, and OXPHOS (CI‐NDUFB8, CII‐SDHB, CIII‐UQCRC2, CIV‐MTCO1, CV‐ATP5A) in primary PTCs from different group cells (*n* = 4). H) Oxygen consumption rate (OCR) of primary PTCs in both the si‐NC and si‐ERRα group (*n* = 3). I) Representative plots and statistical graphs for flow cytometry analysis of MitoTracker‐red in primary PTCs infected with si‐NC or si‐ ERRα and treated with 40 × 10^−3^ m glucose for 24 h. J) Mitochondrial‐related genes expression profiles were compared between different group of HK‐2 cells. Data are presented as mean ± SEM. ns: *p* > 0.05; *p < 0.05; **p < 0.01; ***p < 0.001.

In exploring ERRα’s role in DKD, we utilized two established diabetes models: the db/db mouse model at 16 weeks of age with a BKS genetic background and the STZ‐induced model, created through 16‐week post‐intraperitoneal streptozotocin injection.^[^ ^16^ ^]^ Both models demonstrated a marked reduction of ERRα expression within kidney tissues, compared to their respective controls (db/m mice or those receiving citrate buffer injections), as evident in immunofluorescence and immunohistochemistry analyses (**Figure** **2**A–D; Figure S2A–D, Supporting Information). Further investigation into renal tubules from db/db and db/m mice through mRNA sequencing revealed the substantial suppression of over 170 genes related to mitochondrial biogenesis and architecture, notably PGC1α and TOM20 (Figure S2E,F, Supporting Information). Western blot assays corroborated these findings, indicating a pronounced decrease in mitochondrial‐related proteins, including PGC1α, TOM20, and components of the oxidative phosphorylation system (OXPHOS), specifically CI‐NDUFB8, CII‐SDHB, CIII‐UQCRC2, CIV‐MTCO1, CV‐ATP5A (Figure 2E).

To assess the impact of high glucose (HG) conditions, we subjected cells to a viability assay (CCK‐8) and HG concentration gradients. Results confirmed that a 40 × 10^−3^ m HG concentration did not impair cell viability yet significantly diminished ERRα levels (Figure S2G,H, Supporting Information). PTCs isolated and stimulated with HG showcased a similar reduction in ERRα protein levels alongside inhibited mitochondrial function‐related protein expression (Figure 2F,G). Employing siRNA against ERRα decreased oxygen consumption rates (OCR) in PTCs, indicative of impaired mitochondrial function (Figure 2H,I). This phenomenon was mirrored in HK‐2 cells, a human renal cortical proximal tubular epithelial cell line revealed through mRNA sequencing (Figure 2J) and Western blot analysis (Figure S2I–M, Supporting Information). These findings underscore ERRα’s association with mitochondrial impairment in PTCs, presenting it as a critical factor in DKD pathogenesis.

### 2.3 Conditional Knock‐In of ERRα in PTCs Ameliorates Mitochondrial Injury in DKD

To decipher the protective role of ERRα in PTCs during DKD, our study embarked on generating a conditional knock‐in mouse model. By crossing ERRα^floxed^ mice (ERRα^fl/fl^) with Ggt1‐Cre mice, we successfully introduced ERRα specifically into PTCs, as visually confirmed by TEM with immuno‐gold labeling (Figure S3A, Supporting Information). These ERRα^ptKI^ mice exhibited normal phenotypes and followed expected Mendelian ratios, highlighting the viability of our genetic engineering approach (Figure S3B, Supporting Information).

In‐depth examination revealed a significant elevation of ERRα expression in the PTCs of ERRα^ptKI^ mice, nearly doubling that observed in control group littermates (ERRα^ctrl^ mice: ERRα^fl/fl^; Ggt1cre‐ mice), showcasing the successful conditional expression of ERRα within the target cells (Figure S3C, Supporting Information). This enhancement of ERRα was also visualized through TEM with immune‐gold labeling, providing a direct glimpse into the successful generation of ERRα^ptKI^ mice (Figure S3D, Supporting Information).

**Figure 3:**
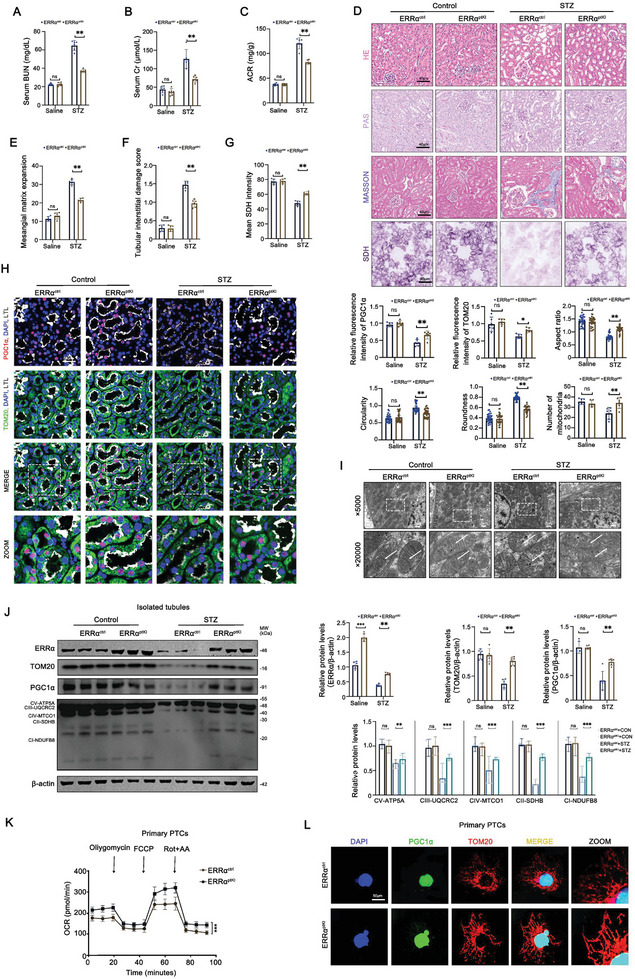
Conditional knock‐in of ERRα in PTCs ameliorates mitochondrial injury in DKD. A–C) Serum urea nitrogen (BUN), serum creatinine (SCr) and urine creatinine protein ratio (ACR) levels in different groups of mice (*n* = 6). D) Representative images of HE, PAS, Masson and SDH stainings from different group of mice (*n* = 6). E) Mesangial matrix expansion of kidney sections in different groups of mice (*n* = 6). F) Tubular interstitial damage score of kidney sections indifferent groups of mice (*n* = 6). G) Mean SDH intensity of kidney sections in different groups of mice (*n* = 6). H) Representative immunofluorescent images of LTL (gray), PGC1α (red), and TOM20 (green) in PTCs from different groups of mice (*n* = 6). I) TEM analyses of PTCs ultrastructure and quantitation of aspect ratio, circularity, roundness in different groups of mice (*n* = 6). The number and area of mitochondria per unit (per ×5000 field of view for mitochondria number). J) Representative Western blot and densitometric analysis of ERRα, TOM20, PGC1α, and OXPHOS (CI‐NDUFB8, CII‐SDHB, CIII‐UQCRC2, CIV‐MTCO1, CV‐ATP5A) in renal tubules from different group mice (*n* = 6). K) Measurement of oxygen consumption rate (OCR) in different groups of primary PTCs (*n* = 3). L) Representative immunofluorescent images of PGC1α (green) and TOM20 (red) of different groups of primary PTCs. Data are presented as mean ± SEM. ns: *P* > 0.05; *p < 0.05; **p < 0.01; ***p < 0.001.

Functionally, this genetic modification led to notable improvements in renal function among DKD models, as evidenced by reductions in blood urea nitrogen (BUN), serum creatinine (SCr), and ACR (**Figure** **3**A–C). Histopathological evaluations through HE, PAS, and Masson staining confirmed significant reductions in tubular atrophy and tubulointerstitial fibrosis. There were no significant changes in the renal function indices as well as pathological staining in the ERRα^ptKI^ mice compared to the control group in the physiological state. However, there was a significant improvement in tubular atrophy and tubulointerstitial fibrosis in ERRα^ptKI^ mice under DKD state. These improvements were coupled with increased succinate dehydrogenase (SDH) activity, indicating enhanced mitochondrial functionality (Figure 3D–G).

Four‐color immunofluorescence (Figure 3H) and immunohistochemistry (Figure 3I) staining for PGC1α, TOM20, and LTL indicated the mitigation of mitochondrial injury in ERRα^ptKI^ mice with DKD. TEM observations corroborated the attenuation of mitochondrial vacuolization, cristae breakage and loss, signifying preserved mitochondrial integrity, as well as a significant reduction in mitochondrial number (Figure S3G, Supporting Information). Western blot analyses further affirmed the mitigation of mitochondrial injury, delineating the ameliorative effect of ERRα overexpression on PTCs in the context of DKD (Figure 3J).

Moreover, OCR measurements from isolated PTCs of ERRα^ptKI^ mice unveiled a significant uptick in basal and maximal respiration, pointing to an invigorated mitochondrial function attributable to ERRα overexpression. This metabolic boost was visually captured through immunofluorescence staining, revealing denser and more vibrant mitochondrial networks alongside elevated PGC1α expression in ERRα^ptKI^ mice compared to controls (Figure 3K–L; Figure S3E,F, Supporting Information).

Collectively, these findings elucidate ERRα’s pivotal role in safeguarding mitochondrial integrity within PTCs amidst DKD, endorsing the strategic overexpression of ERRα as a potential therapeutic avenue for mitigating mitochondrial‐related injuries in DKD.

### 2.4 Overexpression of ERRα Mitigates High Glucose‐Induced Mitochondrial Damage in Renal Cells

To evaluate the cytoprotective potential of ERRα against HG‐induced mitochondrial damage, we employed HK‐2 cells overexpressing ERRα through lentiviral transduction (LV‐ERRα). Our analysis revealed that the LV‐ERRα group demonstrated notable restoration of TOM20, PGC1α, and OXPHOS‐associated proteins, which were otherwise reduced under HG conditions, underscoring the protective role of ERRα in maintaining mitochondrial protein integrity (Figure S4A, Supporting Information). Fluorescence imaging further confirmed enhanced mitochondrial structure and function in LV‐ERRα cells, as evidenced by increased TOM20 staining, indicative of robust mitochondrial integrity and activity (Figure S4B, Supporting Information).

Functional assays, including OCR and ATP production measurements, provided quantitative evidence that ERRα overexpression effectively countered HG‐induced impairments in mitochondrial respiration and ATP synthesis. The LV‐ERRα cells exhibited significantly improved mitochondrial respiratory capacity and ATP generation in the presence of HG, highlighting the efficacy of ERRα in reversing mitochondrial dysfunction (Figure S4C,D, Supporting Information).

Ultrastructural analysis using TEM offered direct visualization of mitochondrial health in HG‐treated LV‐ERRα cells. The results depicted a marked reduction in mitochondrial damage (mitochondrial vacuolization, cristae breakage and loss) compared to control cells subjected to HG stress (Figure S4E, Supporting Information).

Collectively, these findings elucidate the critical role of ERRα overexpression in safeguarding renal cells from HG‐induced mitochondrial distress. By enhancing mitochondrial protein expression, structural integrity, respiratory function, and energy production, ERRα emerges as a key modulator of cellular resilience against metabolic challenges in diabetic conditions. This study highlights ERRα’s protective mechanism and strengthens the rationale for targeting ERRα pathways in therapeutic strategies aimed at mitigating mitochondrial‐related renal damage in DKD.

### 2.5 RBBP6 Mediates Degradation of ERRα through Ubiquitin‐Proteasome System

**Figure 4:**
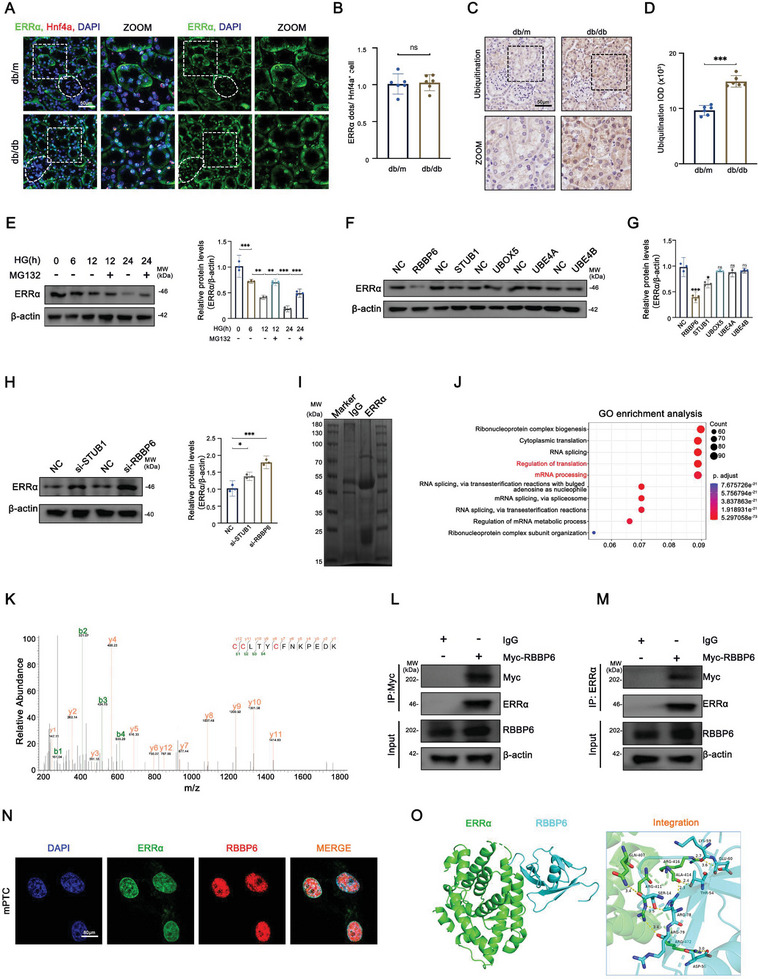
RBBP6 mediates the degradation of ERRα via UPS. A,B) Representative images and quantification of fluorescent in situ hybridization (FISH) for ERRα and Hnf4a in PTCs of db/m and db/db mice (*n* = 6). C,D) Representative immunohistochemistry images and quantitative analysis of ubiquitination in PTCs from db/m and db/db mice (*n* = 6). E) Representative Western blot and densitometric analysis of ERRα in different groups of HK‐2 cells (*n* = 3). F,G) Representative Western blot and densitometric analysis of ERRα in different groups of HK‐2 cells (*n* = 3). H) Representative Western blot and densitometric analysis of ERRα in different groups of HK‐2 cells (*n* = 3). I) SDS‐PAGE gel of proteins bound to IgG (left lane) or ERRα (right lane). Protein marker stands stand for (from top to bottom): 180, 130, 100, 70, 55, 45, 35, 25, 15/10 kDa. J) Gene Ontology (GO) enrichment of proteins bound to ERRα. K) Protein secondary spectrum of peptide segments of ERRα. L,M) HK‐2 cell lysates were subjected to immunoprecipitation (IP) with IgG, Myc, and ERRα antibody, respectively, followed by RBBP6 immunoblotting (IB). N) Representative confocal microscopic images of colocalization of RBBP6 and ERRα in HK‐2 cells. O) Proposed interaction model of RBBP6 binding to ERRα. 3D illustrations of the interaction between RBBP6 and ERRα. Data are presented as mean ± SEM. ns: *P* > 0.05; *p < 0.05; **p < 0.01; ***p < 0.001.

Our observations indicated a specific reduction in ERRα protein levels in PTCs affected by DKD, suggesting a mechanism distinct from transcriptional downregulation. To explore this, we conducted fluorescent in situ hybridization (FISH) using kidney tissues from both control (db/m) and DKD model (db/db) mice. The mRNA levels of ERRα, marked by hepatocyte nuclear factor 4α (Hnf4a) references,^[^ ^17^, ^18^ ^]^ remained unchanged in DKD‐affected PTCs, pointing towards post‐transcriptional regulation as the culprit for ERRα diminution (**Figure** **4**A,B).

Further, immunohistochemical analyses revealed an upsurge in overall ubiquitination within the renal tissues of db/db mice, implicating the UPS in ERRα degradation under hyperglycemic conditions (Figure 4C,D). This hypothesis was substantiated when treatment with MG132, a proteasome inhibitor, in HG‐exposed HK‐2 cells, resulted in ERRα stabilization, thereby confirming UPS's role in ERRα proteolysis (Figure 4E).

Utilizing UbiBrowser, a bioinformatics platform, we predicted 19 E3 ligases potentially responsible for ERRα ubiquitination. Subsequent overexpression experiments identified RBBP6 and STUB1 as key regulators, with RBBP6 exerting a more pronounced effect on ERRα degradation (Figure 4F–H, Figure S5A–C, Supporting Information). Mass spectrometry analysis of ERRα‐associated proteins and Gene Ontology enrichment further spotlighted RBBP6's interaction with ERRα (Figure 4I–K, Figure S5D, Supporting Information). Co‐immunoprecipitation, double immunofluorescence staining, and spatial binding analysis between ERRα and RBBP6 validated their interaction, illustrating RBBP6's pivotal role in mediating ERRα ubiquitination and proteasomal degradation in PTCs under diabetic conditions (Figure 4L–O).

Together, these findings delineate the RBBP6‐mediated ERRα degradation via the UPS in DKD‐affected PTCs. By elucidating the post‐transcriptional regulation of ERRα, our findings unveil a potential therapeutic target within the RBBP6‐ERRα axis for mitigating mitochondrial dysfunction in DKD.

### 2.6 RBBP6 Expression Increases in Diabetic Kidney Disease

**Figure 5:**
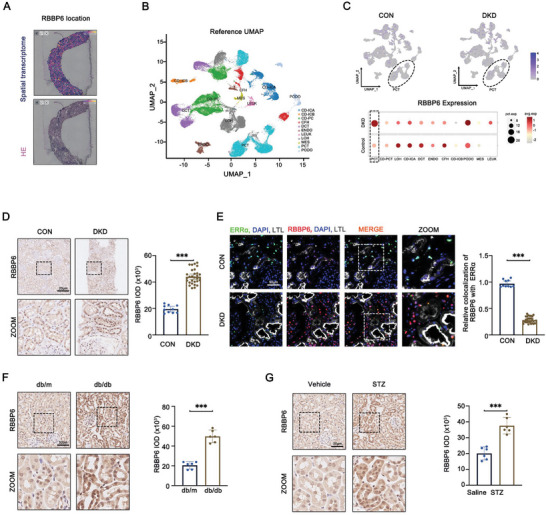
The expression of RBBP6 is upregulated in DKD. A) Spatial transcriptomics for DKD patient (Kidney Tissue Atlas, https://atlas.kpmp.org/, Kidney Tissue Atlas Spatial Transcriptomics for sample S‐2109‐011418, participant 32‐10346). B,C) RBBP6 gene expression in healthy and DKD patients from single‐nucleus RNA‐seq. RBBP6 Expression Comparison across Clusters in healthy and DKD group. (GEO ID: GSE131882). D) Representative immunohistochemistry images and quantification of RBBP6 in PTCs from control (*n* = 10) and DKD (*n* = 30) patients. E) Representative immunofluorescent images of ERRα (green) and RBBP6 (red) of PTCs in control (*n* = 10) and DKD (*n* = 30) patients. F) Representative immunohistochemistry images and quantification of RBBP6 in PTCs of db/m and db/db mice (*n* = 6). G) Representative immunohistochemistry images and quantification of RBBP6 in PTCs of Vehicle and STZ group mice (*n* = 6). Data are presented as mean ± SEM. ***p < 0.001.

Exploring RBBP6's renal distribution, we turned to spatial transcriptomics data from the Kidney Precision Medicine Project and single‐nucleus RNA sequencing data from the Gene Expression Omnibus (GSE131882 and GSE181382). This analysis pinpointed a marked enrichment of RBBP6 mRNA within PTCs, with a notable elevation in DKD conditions (**Figure** **5**A–C, Figure S6A, Supporting Information). Immunohistochemistry reinforced these findings, showing RBBP6's heightened presence in DKD‐affected kidney tissues (Figure 5D).

Further, co‐localization studies of RBBP6 and ERRα in DKD patient samples demonstrated an increase in RBBP6 alongside a decrease in ERRα within PTCs, aligning with the dysregulation pattern observed in DKD animal models (Figure 5E–G; Figure S6B–D, Supporting Information). This collective evidence underscores RBBP6's upregulation as a potential driving force behind mitochondrial impairment in PTCs during DKD progression.

### 2.7 RBBP6 Regulates Mitochondrial Injury in PTCs through ERRα Degradation

**Figure 6:**
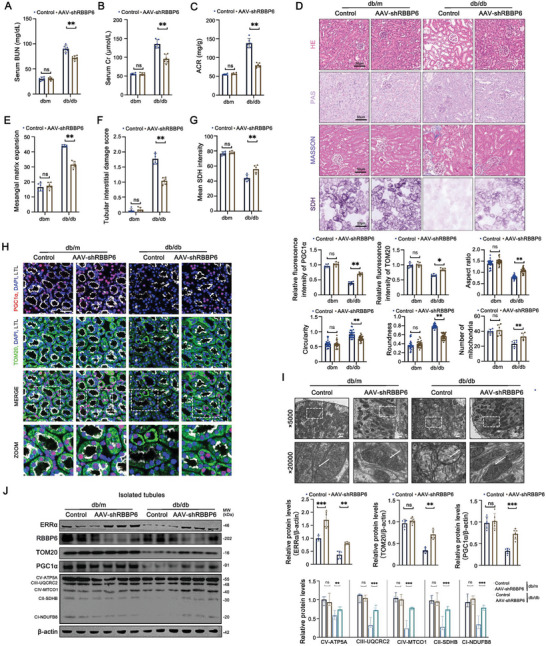
Downregulation of RBBP6 can alleviate pathological damage and ameliorate mitochondrial injury. A–C) Levels of BUN, SCr, and ACR in different groups of mice (*n* = 6). D) Representative images of HE, PAS, Masson and SDH stainings from different groups of mice (*n* = 6). E) Mesangial matrix expansion of kidney sections in different group of mice (*n* = 6). F) Tubular interstitial damage score of kidney sections in different groups of mice (*n* = 6). G) Mean SDH intensity of kidney sections in different groups of mice (*n* = 6). H) Representative immunofluorescent images of LTL (gray), PGC1α (red), and TOM20 (CY5) in PTCs from different groups of mice (*n* = 6). I) TEM of PTCs ultrastructure and quantitation of aspect ratio, circularity, roundness in different groups of mice (*n* = 6). The number and area of mitochondria per unit (per ×5000 field of view for mitochondria number). J) Representative Western blot and densitometric analysis of ERRα, TOM20, PGC1α, and OXPHOS (CI‐NDUFB8, CII‐SDHB, CIII‐UQCRC2, CIV‐MTCO1, CV‐ATP5A) in renal tubules from different groups of mice (*n* = 6). Data are presented as mean ± SEM. ns: *P* > 0.05; *p < 0.05; **p < 0.01; ***p < 0.001.

In our quest to dissect the role of RBBP6 in DKD, we embarked on a novel experimental journey by employing adeno‐associated viruses (AAVs) engineered to silence RBBP6 expression (AAV‐shRBBP6) or to serve as controls (AAV‐Control). These AAVs were specifically delivered to renal tissues of db/db or db/m mice via precise in situ renal injections, as depicted in Figure S7A (Supporting Information). This approach led to a marked reduction, by about 50%, in RBBP6 expression within renal tubular cells of the AAV‐shRBBP6‐treated mice, spotlighting the efficiency of our gene silencing strategy (Figure S7B, Supporting Information). Most compelling was the observation that attenuating RBBP6 expression in db/db mice significantly ameliorated renal function, evident from improved BUN, SCr, and ACR levels (**Figure** **6**A–C). Histological insights gained through HE, PAS, and Masson stainings underscored the therapeutic potential of RBBP6 silencing, revealing a remarkable reduction in tubular atrophy and tubulointerstitial fibrosis, alongside enhanced SDH activity in the AAV‐shRBBP6 group (Figure 6D–G). The alleviation of mitochondrial injury by RBBP6 knockdown was further illuminated through four‐color immunofluorescence and immunohistochemistry staining, capturing a vivid representation of mitochondrial preservation in the treated db/db mice (Figure 6H, Figure S7C, Supporting Information). TEM corroborated these findings, indicating the attenuation of mitochondrial vacuolization, cristae breakage and loss, as well as a significant reduction in mitochondrial number in AAV‐shRBBP6‐treated mice (Figure 6I). Additionally, a significant resurgence in the expression of mitochondrial‐related genes, including PGC1α, TOM20, and OXPHOS components, was documented in the AAV‐shRBBP6 treated db/db mice, delineating a direct link between RBBP6 modulation and mitochondrial functional recovery (Figure 6J).

**Figure 7:**
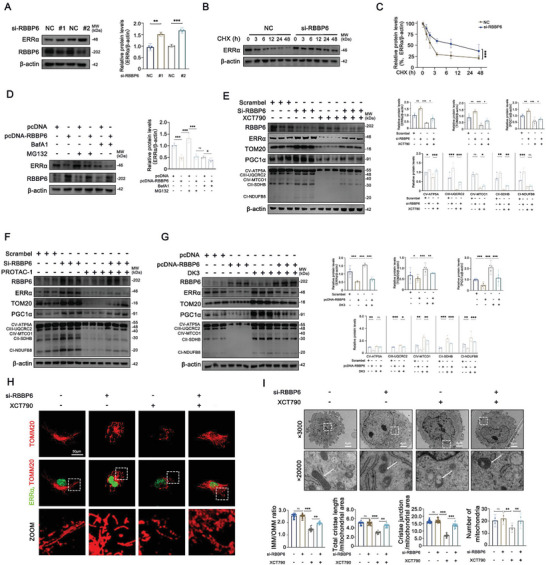
Downregulation of RBBP6 ameliorates mitochondrial injury in HK‐2 cells. A) Representative Western blot and densitometric analysis of ERRα and RBBP6 in HK‐2 cells transfected with si‐RBBP6 or si‐NC (*n* = 3). B,C) Representative Western blot and densitometric analysis of ERRα in HK‐2 cells treated with cycloheximide (CHX) of different concentrations and transfected with si‐RBBP6/si‐NC (*n* = 3). D) Representative Western blot and densitometric analysis of ERRα and RBBP6 in HK‐2 cells treated with MG132/BafA1 and transfected with pcDNA‐RBBP6/pcDNA (*n* = 3). E,F) Representative Western blot and densitometric analysis of ERRα, TOM20, PGC1α, and OXPHOS (CI‐NDUFB8, CII‐SDHB, CIII‐UQCRC2, CIV‐MTCO1, CV‐ATP5A) in HK‐2 cells treated with XCT790/PROTAC‐1 and transfected with si‐RBBP6/si‐NC (*n* = 3). G) Representative Western blot and densitometric analysis of ERRα, TOM20, PGC1α, and OXPHOS (CI‐NDUFB8, CII‐SDHB, CIII‐UQCRC2, CIV‐MTCO1, CV‐ATP5A) in HK‐2 cells treated with DK3 and transfected with pcDNA‐RBBP6/pcDNA (*n* = 3). H) Representative immunofluorescent images of ERRα (green) and TOM20 (red) in HK‐2 cells after transfecting with si‐NC or si‐RBBP6. Cells were either unstimulated or treated with XCT790 (*n* = 3). I) TEM analyses of HK‐2 cell ultrastructure and quantitation of IMM/OMM ratio, total cristae length/mitochondrial area, cristae junction/mitochondrial area in different group. The number and area of mitochondria per unit (per ×5000 field of view for mitochondria number) (*n* = 3). Data are presented as mean ± SEM. *p < 0.05; **p < 0.01; ***p < 0.001.

To extend our investigation into the cellular mechanisms underpinning the observed in vivo effects, we employed HK‐2 cells, a human renal cortical proximal tubular epithelial cell line, as an in vitro model. By silencing RBBP6 via siRNA, we witnessed a notable escalation in ERRα protein levels, suggesting that RBBP6 mediates ERRα degradation post‐transcriptionally (**Figures** **7**A, and S8A, Supporting Information). The application of cycloheximide (CHX), an inhibitor of eukaryotic protein synthesis, further elucidated the protective role of RBBP6 silencing on ERRα stability under HG conditions (Figure 7B,C). Conversely, the overexpression of RBBP6 precipitated a decline in ERRα levels, a process reversibly mitigated by the proteasome inhibitor MG132, highlighting the UPS critical involvement in ERRα regulation under diabetic stressors (Figure 7D). The intricate relationship between RBBP6 and ERRα was further dissected using XCT790 and PROTAC ERRα Degrader‐1 (PROTAC‐1), revealing that modulating RBBP6 activity influences the restoration of mitochondrial function, as evidenced by the regulation of mitochondrial‐related genes (Figure 7E,F; Figures S8B–S7E, Supporting Information). Additionally, mitochondrial health was preserved by the ERRα agonist DK3 in the context of RBBP6 overexpression in HK‐2 cells (Figure 7G). Immunofluorescence and TEM analyses provided a visual confirmation of how targeting ERRα with XCT790 accentuated mitochondrial distress (mitochondrial vacuolization, cristae breakage and loss, as well as a significant reduction in mitochondrial number) (Figure 7H,I), collectively underscoring the pivotal role of RBBP6 in modulating mitochondrial integrity through ERRα degradation in the diabetic milieu.

### 2.8 RBBP6 Promotes K48‐Linked Ubiquitination of ERRα

**Figure 8:**
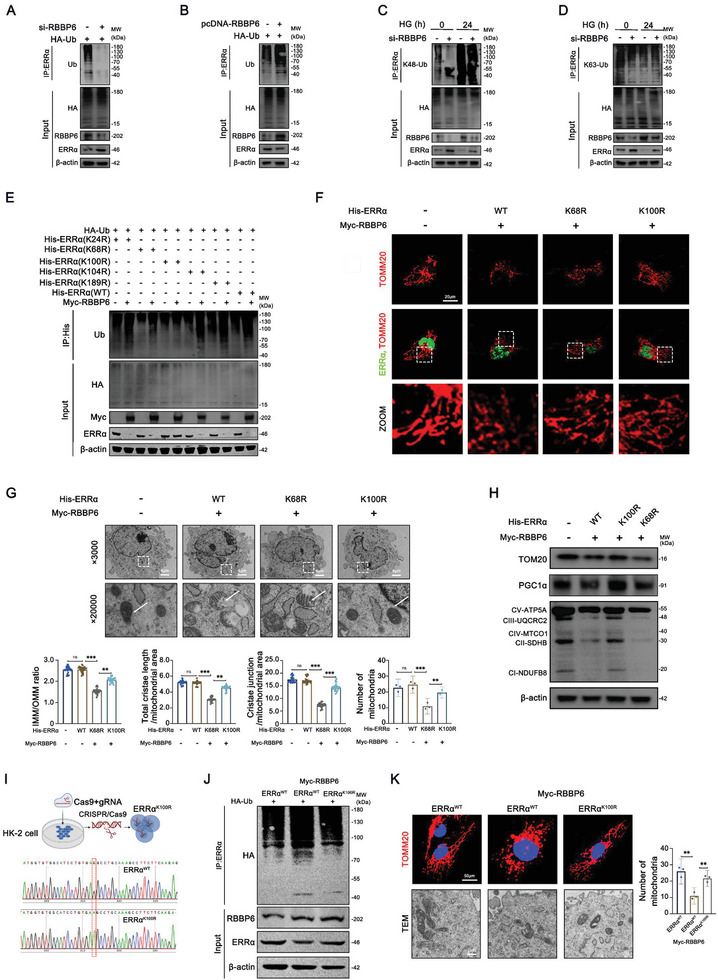
RBBP6 promotes K48‐linked ubiquitination at the K100 residue of ERRα. A,B) HK‐2 cells were transfected with si‐RBBP6 or pcDNA‐RBBP6, and HA‐ubiquitin for 6 hours and incubated with antibiotic‐free medium. Cell lysates were subjected to IP with ERRα antibody, followed by HA, RBBP6, and ERRα (IB) (*n* = 3). C,D) HK‐2 cells were transfected with si‐RBBP6 for 6 h and incubated with antibiotic‐free medium and 40 × 10^−3^ m HG stimulation for an additional 24 h. Cell lysates were subjected to IP with ERRα antibody, followed by K48‐ubiquitin/K63‐ubiquitin, RBBP6, ERRα (IB) (*n* = 3). E) HK‐2 cells were transfected with HA‐ubiquitin, Myc‐RBBP6 plasmid and different mutant ERRα plasmids, and the immunoprecipitation with His affinity gels and immunoblotting were performed (*n* = 3). F) Representative immunofluorescent images of ERRα (green) and TOM20 (red) in HK‐2 cells transfected with Myc‐RBBP6 plasmid, different mutant ERRα plasmids (K68 and K100 residues) (*n* = 3). G) HK‐2 cells were transfected with Myc‐RBBP6 plasmid and different mutant ERRα plasmids (K68 and K100 residues). TEM analyses of HK‐2 cell ultrastructure and quantitation of IMM/OMM ratio, total cristae length/mitochondrial area, cristae junction/mitochondrial area in different groups. The number and area of mitochondria per unit (per ×5000 field of view for mitochondria number) (*n* = 3). H) Representative Western blot of ERRα, TOM20, PGC1α, and OXPHOS (CI‐NDUFB8, CII‐SDHB, CIII‐UQCRC2, CIV‐MTCO1, CV‐ATP5A) in HK‐2 cells from different group (*n* = 3). I) Pattern map and Sanger sequencing results of ERRα K100 site mutated to arginine (R) in HK‐2 cells. J) Representative Western blot of RBBP6 and ERRα in HK‐2 cells from different group (*n* = 3). K) Representative immunofluorescent images of TOM20 and TEM analyses of HK‐2 cell ultrastructure and quantitation. The number and area of mitochondria per unit (per ×5000 field of view for mitochondria number) (*n* = 3). Data are presented as mean ± SEM. ns: *p* > 0.05; **p < 0.01; ***p < 0.001.

To explore the specific molecular dynamics between RBBP6 and ERRα in DKD, we engaged in a series of cellular experiments utilizing HK‐2 cells. Initial assays involved transfecting these cells with plasmids expressing HA‐tagged ubiquitin. The presence of RBBP6 siRNA resulted in a notable suppression of ubiquitinated protein levels, underscoring the crucial role of RBBP6 in the ubiquitination process. This suppression was further confirmed by Western blot analysis of immunoprecipitated ERRα, revealing a marked inhibition of ERRα ubiquitination upon RBBP6 knockdown (**Figure** **8**A). Conversely, RBBP6 overexpression led to enhanced ubiquitination of ERRα (Figure 8B), highlighting the enzyme's pivotal function in the UPS.

Given the diversity of ubiquitin chain types in mammalian cells (specifically, K48‐ and K63‐linked chains that dictate distinct cellular fates for substrate proteins), we investigated the specific type of ubiquitination mediated by RBBP6. Through employing mutant HA‐tagged ubiquitin plasmids (either K48 or K63), our studies indicated a significant diminution in K48‐linked ubiquitination levels of ERRα with RBBP6 siRNA, while K63‐linked ubiquitination remained unchanged.^[^ ^19^ ^]^ This delineates RBBP6's role in facilitating K48‐linked ubiquitination, directing ERRα towards proteasomal degradation.

To identify critical lysine residues on ERRα subjected to ubiquitination, we utilized the BDM‐PUB database for predictions, focusing on high‐confidence sites (Figure S9A, Supporting Information). Mutational analysis of these sites (His‐tagged ERRα in which the relevant lysine residues were individually mutated to arginine) revealed the indispensable nature of the K100 residue for RBBP6‐mediated ubiquitination of ERRα, particularly under HG conditions, as confirmed by co‐immunoprecipitation and subsequent analysis (Figure 8E and Figure S9B, Supporting Information).

The downstream effects of this ubiquitination were evident in the restoration of mitochondrial integrity upon mutation of ERRα K100 residue in PTCs and HK‐2 cells, as demonstrated through immunofluorescence staining (Figure 8F), TEM (Figure 8G, Figure S9C, Supporting Information), and Western blot analysis (Figure 8H, Figure S9D, Supporting Information). To further validate the role of K100 in ERRα, we also mutated the K100 site using CRISPR/Cas9 technology, and consistent with previous results, mutation of K100 to arginine reversed the degradation of ERRα and significantly ameliorated the mitochondrial damage (mitochondrial swelling, mitochondrial cristae breakage and disappearance, and reduction in mitochondrial number) (Figure 8I–K). This restoration was corroborated in vivo studies, where AAV‐mediated introduction of ERRα with a mutated K100 residue (ERRα^K100R^) significantly improved renal function and attenuated mitochondrial damage in DKD models, as evidenced by histological and biochemical analyses (Figure S10A–I, Supporting Information).

These findings collectively illuminate RBBP6's role in promoting K48‐linked polyubiquitination of ERRα at the K100 residue, a process pivotal to regulating mitochondrial injury in proximal tubular cells under diabetic conditions. This enriches our understanding of DKD pathophysiology and unveils a potential therapeutic target for mitigating disease progression.

## 3 Discussion

**Figure 9:**
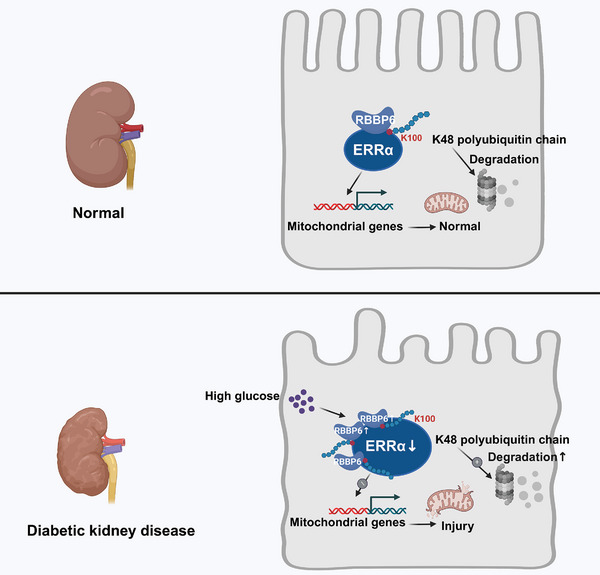
Downregulation of ERRα and mitochondrial impairment in diabetic kidney disease.

Mitochondrial dysfunction is increasingly recognized as a pivotal factor in DKD pathogenesis, with the regulation of ERRα playing a crucial role.^[^ ^5^ ^]^ Our research unveils that in PTCs affected by DKD, there is a notable downregulation of ERRα, mediated by the UPS. We discovered that RBBP6 directly interacts with ERRα, facilitating its K48‐linked polyubiquitination at the K100 residue, which leads to ERRα’s degradation (**Figure** **9**). This reduction in ERRα levels is associated with mitochondrial damage in PTCs, contributing to the worsening of DKD. This insight into the molecular dynamics between RBBP6 and ERRα underscores the critical role of mitochondrial health in DKD progression and opens new avenues for therapeutic interventions targeting these interactions.

The primary function of PTCs is reabsorption, which demands a substantial energy supply predominantly provided by mitochondria, the cell's powerhouses. This study corroborates existing research^[^ ^20^, ^21^, ^22^, ^23^ ^]^ indicating that mitochondrial dysfunction marked by impaired quality control, altered metabolism and dynamics, and elevated oxidative stress plays a pivotal role in the onset and progression of renal pathologies, including acute kidney injury, fibrosis, and notably, DKD. Specifically, our findings reveal pronounced mitochondrial damage within PTCs during DKD's advancement, evident through significant mitochondrial swelling and structural loss, thus underscoring the critical impact of mitochondrial health on renal disease progression.

The etiology of DKD remains multifaceted and not fully understood. Among the hypothesized mechanisms contributing to its pathogenesis are the accumulation of advanced glycation end products,^[^ ^24^ ^]^ activation of protein kinase C pathways,^[^ ^25^ ^]^ and enhanced oxidative stress.^[^ ^26^ ^]^ These factors are known to inflict mitochondrial damage, thereby accelerating DKD progression.^[^ ^27^ ^]^ Our investigations, encompassing both clinical and animal model studies, have substantiated significant mitochondrial dysfunction within PTCs in DKD, characterized by noticeable swelling and structural disruption of mitochondria. This concurs with existing literature, reinforcing the central role of mitochondrial integrity in the pathophysiology of DKD.

ERRα, pivotal in cellular metabolism and mitochondrial integrity, stands at the forefront of research into metabolic disorders.^[^ ^28^, ^29^ ^]^ This transcription factor's expression varies across diseases, with increased levels linked to poor outcomes in several cancers, including breast cancer, where it correlates with osteoporosis.^[^ ^30^, ^31^ ^]^ Conversely, diminished ERRα expression has been implicated in vascular inflammation,^[^ ^32^ ^]^ acute lung injury,^[^ ^33^ ^]^ Alzheimer's disease,^[^ ^34^ ^]^ and heart failure,^[^ ^35^ ^]^ highlighting its broad physiological relevance. In type 2 diabetes, ERRα exhibits organ‐specific expression patterns, with reductions in skeletal muscle and elevations in the liver.^[^ ^8^, ^36^ ^]^ This variability extends to renal pathologies; for example, ERRα deficiency was shown to intensify tubular and mitochondrial damage in a model of cisplatin‐induced acute kidney injury,^[^ ^37^ ^]^ while its expression increased in nephrotic syndrome induced by puromycin aminonucleoside but decreased in PTCs.^[^ ^38^, ^39^ ^]^ Our comparative study of ERRα levels in PTCs between DKD patients and those with non‐DKD renal diseases confirms a specific reduction in ERRα within DKD contexts, underscoring its significant yet nuanced role in DKD's pathological landscape.

Both prior and our investigations have illuminated the association between ERRα and the preservation of mitochondrial integrity and the mitigation of mitochondrial injury. Nonetheless, the exact mechanism remains elusive.^[^ ^40^ ^]^ Recent chromatin immunoprecipitation and deep sequencing (ChIP‐seq) analyses have revealed ERRα’s binding to over one hundred gene‐regulated regions of the mitochondrial electron transport chain, indicating that compromised ERRα functionality results in diminished mitochondrial integrity, activity, and ATP production.^[^ ^14^ ^]^ Moreover, recent studies have demonstrated the influence of targeting ERRα on mitochondrial oxidative phosphorylation, thereby regulating mitochondrial damage.^[^ ^41^ ^]^ Concurrently, increased oxidative stress within mitochondria leads to elevated reactive oxygen species (ROS) production, which in turn causes mitochondrial injury. In this context, ERRα functions as a sensor.^[^ ^42^ ^]^ Conversely, cells can neutralize toxins produced by mitochondrial damage by activating adenosine monophosphate‐activated protein kinase (AMPK). Notably, AMPK activation substantially enhances ERRα expression, promoting mitochondrial biogenesis.^[^ ^43^ ^]^ Additionally, mitophagy, crucial for removing damaged mitochondria, is mediated by ERRα in PTCs.^[^ ^44^ ^]^ While ERRα co‐activates with PGC1α under normal physiological conditions, it's noteworthy that tissues exhibiting high PGC1α expression also display increased ERRα levels, suggesting a potential link between ERRα’s role in reducing mitochondrial injury and PGC1α’s function.^[^ ^45^ ^]^ Our findings underscore alterations in ERRα lead to the impairment of the mitochondrial respiratory chain (OXPHOS) and PGC1α, highlighting these pathways' potential role in mediating ERRα’s mitigation of mitochondrial injury in DKD.

ERRα, a pivotal metabolic regulator and ROS sensor exhibits sensitivity to various external stimuli. In vitro experiments have demonstrated that elevated salt levels^[^ ^46^ ^]^ and fatty acids^[^ ^47^ ^]^ can significantly influence ERRα concentrations, primarily through transcriptional regulation mechanisms. Moreover, recent findings have emphasized the critical role of post‐translational modifications in the pathogenesis of diseases involving ERRα. Notably, carnosic acid has been identified as an inhibitor of osteoporosis by targeting the ubiquitin‐mediated degradation pathway of ERRα.^[^ ^48^ ^]^ Similarly, FBXL10 has been shown to enhance the mono‐ubiquitination of ERRα, thereby fostering the proliferation of breast cancer cells.^[^ ^49^ ^]^ Additionally, the mTOR pathway has been implicated in the modulation of ERRα activity via the UPS, affecting the progression of nonalcoholic fatty liver disease (NAFLD).^[^ ^13^ ^]^

In our study, HG stimulation resulted in a marked reduction in ERRα protein levels without altering mRNA expression, a phenomenon reversible by the proteasome inhibitor MG132. This suggests that the primary regulation of ERRα under diabetic conditions may be attributed to protein stabilization mechanisms, with the UPS playing a central role. Specifically, in DKD, HG exposure appears to enhance proteasome activity, thereby intensifying UPS involvement.^[^ ^50^ ^]^ In contrast, inhibition of the proteasome offers protective benefits in DKD, indicating that dysregulated UPS function could be crucial in its pathogenesis.^[^ ^51^ ^]^

Through mass spectrometry analysis, RBBP6 was identified as a novel E3 ubiquitin ligase for ERRα, highlighting its role in the ubiquitination and subsequent degradation of specific substrates, such as IκBα,^[^ ^52^ ^]^ ZBTB38,^[^ ^53^ ^]^ and YB‐1.^[^ ^54^ ^]^ Elevated expression of RBBP6, associated with poor prognosis in various malignancies,^[^ ^55^ ^]^ was observed in DKD conditions, co‐localizing with ERRα in the nucleus of PTCs. This interaction between RBBP6 and ERRα facilitates the accelerated degradation of ERRα via UPS, leading to mitochondrial damage under diabetic stress.^[^ ^56^, ^57^ ^]^ Our findings further delineate the K100 residue of ERRα as a critical site targeted by RBBP6 for ubiquitination. Intriguingly, mutations at ERRα’s ubiquitination sites prevented its UPS‐mediated degradation and mitigated mitochondrial damage, presenting a novel therapeutic avenue for DKD management.

In conclusion, our research underscores the critical protective role of ERRα in PTCs of DKD. We have documented a notable down‐regulation of ERRα in DKD, correlating significantly with mitochondrial damage in PTCs. Importantly, our study elucidates how RBBP6 influences ERRα stability via the UPS, marking a potential therapeutic intervention point for DKD.

DKD represents a multifaceted condition that complicates in vitro modeling, highlighting the necessity for further detailed exploration to illuminate the intricate mechanics by which ERRα influences mitochondrial functionality. Moreover, additional research is imperative to decode the specific mechanisms driving the upregulation of RBBP6 in DKD, providing deeper insights into its pathophysiology and uncovering new avenues for intervention.

## 4 Experimental Section

### Collection of Human Kidney Samples

Renal biopsy samples were collected as a standard component of the clinical diagnostic procedure, detailed in Table S1 (Supporting Information). These specimens were sourced from the nephrology and pathology departments at Renmin Hospital of Wuhan University. Control kidney samples were derived from non‐diseased regions adjacent to cancerous tissues. The study was conducted in strict adherence to the ethical guidelines laid out in the Declaration of Helsinki and was approved by the Research Ethics Committee of Renmin Hospital of Wuhan University Hospital (WDRY2021‐KS034). Informed consent was obtained from all study participants before inclusion.

### Generation of Conditional ERRα Knock‐In Mouse Strains (ERRα^ptKI^)

ERRα^flox/flox^ knock‐in mice on a C57BL/6N genetic background were acquired from Cytogenes Biosciences (Suzhou, China). Utilizing the CRISPR/Cas9 system, we constructed the pCAG‐loxp‐STOP‐loxp‐ERRα vector, which includes the pCAG promoter, the ERRα coding sequence, and a floxed STOP cassette to prevent transcription. The resulting vector was microinjected into the male pronuclei of fertilized mouse eggs. Ggt1‐Cre recombinase expression was used to generate recombinant transgenic offspring (Cygen Biosciences, China; Catalog #: C001028; Genetic Background: C57BL/6N), yielding the F1 generation with targeted ERRα expression. The generation and characterization of these mice were performed under blinded conditions. All animal procedures followed the NIH Guidelines for the Care and Use of Laboratory Animals (Revised 2011) and received ethical clearance from the Animal Use Ethical Committee of Renmin Hospital of Wuhan University, China (WDRM‐20220205).

### Culture and Treatment of HK‐2 Cells

Human kidney 2 (HK‐2) cells, catalog number CL‐0109, were obtained from the Wuhan Procell Cell Bank, China, and cultured in DMEM‐F12 medium supplemented with 10% fetal bovine serum (FBS), maintained at 37 °C in an atmosphere of 5% CO2. The cell line's authenticity was confirmed via short tandem repeat (STR) profiling. Gene silencing experiments involved transfecting cells with siRNAs targeting RBBP6, STUB1, and ERRα using HiPerFect transfection reagent (Qiagen, Germany), per manufacturer instructions. After transfection, cells were treated with 40 × 10^−3^ m high glucose (HG) or control solution for 24 hours. Additionally, plasmid transfections to overexpress RBBP6, STUB1, UBOX5, UBE4A, and UBE4B were conducted using Lipo6000 transfection reagent (Beyotime, China). Drug intervention studies involved exposing treated HK‐2 cells to 20 × 10^−6^ m cycloheximide (CHX, MCE, China) for various durations (0, 3, 6, 12, 24, and 48 h); 100  × 10^−6^ m Bafilomycin A1 (BafA1, MCE, China) or 10 × 10^−6^ m MG132 (MCE, China) for 6 h; and 10 × 10^−6^ m XCT790 or 10 × 10^−6^ m DK3 (both from MCE, China) for 48 h, to investigate protein stability and cellular responses.

The materials and methods used in this study have been described in detail in the Supporting Information.

## Conflict of Interest

The authors declare no conflict of interest.

## Author Contributions

H.H., J.H., and Z.C. contributed equally to this work. H.H. designed all the experiments and performed imaging, cell culture, biochemistry, Seahorse experiments, lentivirus production, and data analysis. J.H. performed cell culture, and RNA‐seq analyses and helped with immunoblots. Z.C., Z.P., Z.Z. and Y.C. helped with cell culture. K.Y. and Z.Z. helped with experimental design. Y.H. and W.L. helped with the animal experiments. X.S. and F.Z. helped complete the TEM analysis. Q.C., G.D., and W.L. supervised the project and provided fundings. All data were generated inhouse, and no paper mill was used. All authors agree to be accountable for all aspects of work ensuring integrity and accuracy.

## Supporting information



Supporting Information

## Data Availability

The data that support the findings of this study are available from the corresponding author upon reasonable request.
