# Supplementary material for: RBBP6‐Mediated ERRα Degradation Contributes to Mitochondrial Injury in Renal Tubular Cells in Diabetic Kidney Disease
Source: Adv Sci (Weinh). 2024 Oct 23;11(46):2405153. doi: 10.1002/advs.202405153 (PMC11633482; doi:10.1002/advs.202405153)
Supplement: Supplementary file 1 — Supporting Information [file ADVS-11-2405153-s001.docx]

**Experimental Section**

**Supplementary complete materials and methods**

**1. Transgenic DKD mice**

Male db/db and db/m mice (8 weeks old) with a BKS genetic background were obtained from Cytogenes Biosciences (Suzhou, China). The mice were then raised for 24 weeks.

**2. Intrarenal adeno-associated virus (AAV) delivery**

Following a previously described method [1], the intrarenal administration of AAV effectively induced the knockdown of RBBP6 *in vivo*. For this purpose, 1 × 10^12^ genomic particles of AAV-CMV-RBBP6 (AAV-shRBBP6) or AAV-CMV-null (AAV-Control) obtained from HANBIO Technology (Shanghai, China) were injected into the kidneys of 16-week-old db/db mice. The injection was performed at six distinct locations through in situ renal injection.

**3. Pharmaceutical treatment**

Male ERRα^ptKI^ mice (8 weeks old) and wild-type C57BL/6J mice (8 weeks old, male) obtained from Cygen Biosciences (Suzhou, China) were subjected to Streptozotocin (STZ) treatment. STZ was administered intraperitoneally at a dose of 65 mg/kg daily for three consecutive days [2]. Diabetes was defined by blood glucose levels exceeding 16.7 mmol/L. Monthly assessments were conducted to monitor mouse body weight and blood glucose levels. After 24 hours, urine samples were collected using metabolic cages to determine the ACR. Blood samples were drawn from the mice to measure BUN and SCr. Eight weeks after STZ injection, all mice were euthanized, and kidney samples were collected for biochemical and pathological examination. All animal experiments were conducted following the guidelines approved by the Research Ethics Committee of the People's Hospital at Wuhan University (20220205). All of the animal care and experimental procedures complied with the guidelines for the Care and Use of Laboratory Animals published by the United States National Institutes of Health (NIH publication, 2011Revision).

**4. Primary PTC culture and intervention**

Primary PTCs were isolated from C57BL/6J mice and ERRαptKI mice. In short, the kidney tissue was taken under aseptic conditions and washed 2-3 times in PBS solution. The renal cortex was then minced, ground in an 80 μm stainless steel mesh sieve, and thoroughly rinsed with PBS solution. The liquid underneath the mesh was poured onto a 150 μm stainless steel mesh sieve, collected, and washed with PBS. The supernatant was discarded after centrifuging the mesh for 5 minutes at 1000 r/min [3]. Immunofluorescence using Cytokeratin 18 (CK18) was used to confirm the identity of the PTCs. Subsequently, the PTCs were stimulated with a concentration of 40 mM HG or a control buffer for 24 hours.

**5. Antibodies**

The following antibodies were utilized: anti-PGC1α (NBP1-04676) antibody was acquired from Novus Biologicals; Anti-OXPHOS (45-8099) antibody was acquired from Thermo Scientific. Anti-HA tag (3724), anti-His tag (12698), anti-MYC tag (2278S), anti-ubiquitin (3936s), anti-K48-linked ubiquitin (8081s) and anti-K63-linked ubiquitin (5621s) antibodies were obtained from Cell Signaling Technology; anti-RBBP6 (ab237514) antibody and anti-ERRα antibody were purchased from Abcam and Genetex respectively; Secondary antibodies including goat anti-mouse IgG-HRP (sc-2005) and goat anti-rabbit IgG-HRP (sc-2004) were prepared by Santa Cruz Biotechnology; Anti-β-actin (20536-1-AP) antibody was ordered from Proteintech. Fluorescence secondary antibodies, including Alexa Fluor 488-labeled goat anti-rabbit IgG antibody (A11008), Alexa Fluor 594-labeled goat anti-rabbit IgG antibody (A-11012), and Alexa Fluor 647-labeled goat anti-rabbit IgG antibody (A-21245) were procured from Invitrogen.

**6. Western blot assay**

Cells and kidney tubular tissues were lysed in RIPA buffer (Cell Signaling Technology, 9806) containing a protease inhibitor mixture (Bimake, B14002-1). The lysates were then separated using SDS-PAGE. The protein concentration was measured using the BCA Protein Assay Kit (Catalogue No. 23227, ThermoFisher). Equivalent samples were incubated with specific primary antibodies, followed by the addition of HRP-labeled secondary antibodies (1:5,000). The bands were scanned using the ChemiDocTM MP imaging system (Bio-Rad, USA).

**7. Histological and immunohistochemical staining in kidney tissues**

At the end of the specified experiments, the mice were euthanized, and their kidneys were perfused with a cold PBS solution before being fixed in 4% formalin at 4°C. Subsequently, the kidney samples were processed using standard protocols for paraffin embedding and serial sectioning at a thickness of 5.0 μm. Masson, HE, PAS, and PASM staining techniques were employed. Immunohistochemical staining was performed on the tissue sections by incubating them with the corresponding primary antibodies overnight at 4°C. Microscopy images were acquired using the scanning microscope (PANNORAMIC SCAN II, 3DHISTECH).

**8. Immunofluorescence staining in cells and kidney tissues**

Following processing, the cells or tissues were fixed with 4% formalin and subsequently blocked using 5% BSA. The appropriate primary antibody was then incubated overnight at 4°C, followed by a 60-minute incubation period with the corresponding secondary antibody. After cleaning the samples, DAPI was utilized for nuclei counterstaining. The resulting fluorescence images of the tissues and cells were examined and acquired using a confocal laser microscope (FV3000, Olympus).

**9. OCR measurement**

Mitochondrial oxygen consumption was evaluated utilizing a mitochondrial stress test kit (103015100, Agilent). OCR was measured utilizing the seahorse Bioscience XFe96 extracellular flux analyzer (XFe96, seahorse Bioscience), as previously described [4].

**10. TEM**

To assess the mitochondrial morphology in mouse renal cortex and cultured cells, tissues were fixed in 2.5% glutaraldehyde, followed by fixation in 2% osmium tetroxide. After dehydration in a series of graded alcohols, the samples were embedded in EPON. Double staining of the sections with uranyl acetate and lead citrate was carried out for 2 hours at room temperature. The sections were then examined and photographed using a Hitachi transmission electron microscope (HT7800) [5].

**11. Real-time quantitative PCR**

Total RNA was isolated from mouse kidney tissues using the Power SYBR^™^ Green Cells-to-CT^™^ kit (4402954, ThermoFisher). Real-time quantitative PCR (RT-qPCR) was performed using the established method [6]. The primer sequences for qPCR are provided in Table S2.

**12. SDH staining**

Succinate Dehydrogenase Staining Kit (Tetrazolium Salt Method) uses succinate as a substrate, which is dehydrogenated under the action of the enzyme. Nitroblue tetrazolium (NBT) is a hydrogen acceptor, which accepts the hydrogen and is reduced to methoxylate, which is bluish-purple in colour, and is quantified to represent the activity of succinate dehydrogenase. In this study, the SDH staining was conducted following the guidelines provided by the manufacturer (Solarbio, China). Briefly, frozen tissue sections with a thickness of 6 μm were prepared. NBT working solution was added, and the sections were incubated at 37°C for 20 minutes. After rinsing with distilled water, the sections were mounted with neutral gum and examined under a microscope for photography. Mean SDH intensity was measured by ImageJ.

**13. Mitochondrial localization**

To locate mitochondria in cells, MitoTracker (M7512, ThermoFisher) was employed following the established protocol, and images were captured using a scanning microscope (PANNORAMIC SCAN II, 3DHISTECH) [7].

**14. Immunoprecipitation**

Protein samples were incubated with antibodies (1-4 µg/ml) overnight at 4°C. Subsequently, the samples were washed three times with RIPA lysis buffer. Protein A/G agarose beads (Thermo, 20,421) were added to the antibody-protein complexes and incubated for 4 hours at 4°C. In the case of tagged proteins, affinity gels (Bimake, B23102) were directly mixed with the protein lysate and washed three times with RIPA lysis buffer, followed by an overnight incubation at 4°C. After centrifugation, the precipitated fractions were rinsed four times with RIPA lysis buffer and resuspended in 50 µl of 2× SDS-PAGE buffer. The suspension was then boiled at 100°C for 10 minutes, centrifuged, and the supernatant was subjected to Western blots or mass spectrometry to analyze co-precipitated proteins or target protein modifications [8]. The LC-MS/MS analysis was performed by SpecAlly Life Technology Co.,Ltd.(Wuhan, China).

**15. mRNA sequencing**

Total RNA was extracted from kidney tissues and cells using the Trizol reagent (Invitrogen, USA). The concentration of RNA was measured using a NanoDrop spectrophotometer (NanoDrop One, ThermoFisher). mRNA sequencing analysis was conducted by Novogene (China). DESeq2 was employed to identify differentially expressed genes (adjusted p < 0.05). The heatmap of the differentially expressed genes discovered in our study was generated using TB tools [9].

**16. Protein-protein docking**

The activity of protein-protein binding was validated through molecular docking. For this study, the molecular docking process was conducted utilizing the HDOCK online website (http://hdock.phys.hust.edu.cn/). HDOCK evaluates the binding activity of protein-protein docking in different conformations, focusing on amino acid residues within 5Å of interaction distance. The structures of human docking proteins ERRα (PDBID: 1xb7) and RBBP6 (PDBID: 2c7h) were obtained from the RCSB protein database. The PyMOL software (version 4.3.0, https://pymol.org/) was utilized for the separation of the original ligand and protein structures, dehydration, and removal of organics. Ligplus software was employed to investigate the contact force between the two proteins at a two-dimensional angle. Additionally, the PyMOL program (version 4.3.0) was used to map the interaction between amino acid residues.

**17. Fluorescent in situ hybridization (FISH)**

As previously described, FISH was conducted following the manufacturer's protocol (F32951, Invitrogen) [10]. ERRα and Hnf4a sections were hybridized at room temperature for 2 hours, after which they were incubated with fluorescent probes specific to the target probe RNA. Images were acquired utilizing a scanning microscope (PANNORAMIC SCAN II, 3DHISTECH).

**18.** **Cell viability assay**

The CCK-8 assay (ab228554, abcam) was used to detect the effects of different glucose concentrations on HK-2 cell viability. Cells were seeded in 96-well plates at a concentration of 2×10^3^ cells/well and then treated with different concentrations of drugs. After treatment, 10 μl CCK-8 solution was added to each well. After incubation, absorbance was recorded at 450 nm using a Perkin Elmer microplate reader and incubated for 2 h at 37°C under dark conditions.

**19. Construction of ERRα mutants in HK-2 cells**

The ERRα^K100R^ mutants in HK-2 cells were all produced by WZBio (Wuhan, China). Briefly, the ribonucleoprotein (RNP) complex composed of Cas9 protein and gRNA (gRNA: CTATGGTGTGTGGCATCCTGTG-AGG) and the ERRα mutant Oligo sequences were transfected into HK-2 cells by electroporation, and then the CRISPR/Cas9-mediated ERRα mutant monoclonal cells were obtained by PCR screening and finally Sanger sequencing was used for mutation verification.

**20. Statistical analysis**

The quantitative data were presented as mean ± SEM. Statistical analyses were conducted using GraphPad Prism 9.0 software (GraphPad Software, USA). T-tests were employed for comparisons between two groups, while one-way ANOVA followed by Tukey's multiple comparison test was used for statistical comparisons among more than two groups. Each experiment was repeated at least three times. Bivariate correlation analysis was performed using Pearson and Spearman rank correlation analysis. Differences were considered significant at p < 0.05.

**References**

1. Zhan P, Zhang Y, Shi W, et al. Myeloid-derived growth factor deficiency exacerbates mitotic catastrophe of podocytes in glomerular disease. *Kidney International*. 2022;102(3):546-559. doi:10.1016/j.kint.2022.04.027

2. Yang X, Chen Z, Luo Z, et al. STING deletion alleviates podocyte injury through suppressing inflammation by targeting NLRP3 in diabetic kidney disease. *Cellular Signalling*. 2023;109:110777. doi:10.1016/j.cellsig.2023.110777

3. Boogaard PJ, Nagelkerke JF, Mulder GJ. Renal proximal tubular cells in suspension or in primary culture as in vitro models to study nephrotoxicity. *Chemico-Biological Interactions*. 1990;76(3):251-291. doi:10.1016/0009-2797(90)90096-6

4. Chen Z, Zhu Z, Liang W, et al. Reduction of anaerobic glycolysis contributes to angiotensin II-induced podocyte injury with foot process effacement. *Kidney International*. 2023;103(4):735-748. doi:10.1016/j.kint.2023.01.007

5. Zhang Z, Liang W, Luo Q, et al. PFKP Activation Ameliorates Foot Process Fusion in Podocytes in Diabetic Kidney Disease. *Front Endocrinol*. 2022;12:797025. doi:10.3389/fendo.2021.797025

6. Luo Q, Liang W, Zhang Z, et al. Compromised glycolysis contributes to foot process fusion of podocytes in diabetic kidney disease: Role of ornithine catabolism. *Metabolism*. 2022;134:155245. doi:10.1016/j.metabol.2022.155245

7. Sun H, Li H, Yan J, et al. Loss of CLDN5 in podocytes deregulates WIF1 to activate WNT signaling and contributes to kidney disease. *Nat Commun*. 2022;13(1):1600. doi:10.1038/s41467-022-29277-6

8. Feng J, Chen Z, Ma Y, et al. AKAP1 contributes to impaired mtDNA replication and mitochondrial dysfunction in podocytes of diabetic kidney disease. *Int J Biol Sci*. 2022;18(10):4026-4042. doi:10.7150/ijbs.73493

9. Chen C, Chen H, Zhang Y, et al. TBtools: An Integrative Toolkit Developed for Interactive Analyses of Big Biological Data. *Molecular Plant*. 2020;13(8):1194-1202. doi:10.1016/j.molp.2020.06.009

10. Taguchi K, Elias BC, Sugahara S, et al. Cyclin G1 induces maladaptive proximal tubule cell dedifferentiation and renal fibrosis through CDK5 activation. *Journal of Clinical Investigation*. 2022;132(23):e158096. doi:10.1172/JCI158096

**Supplemental Figures**

**
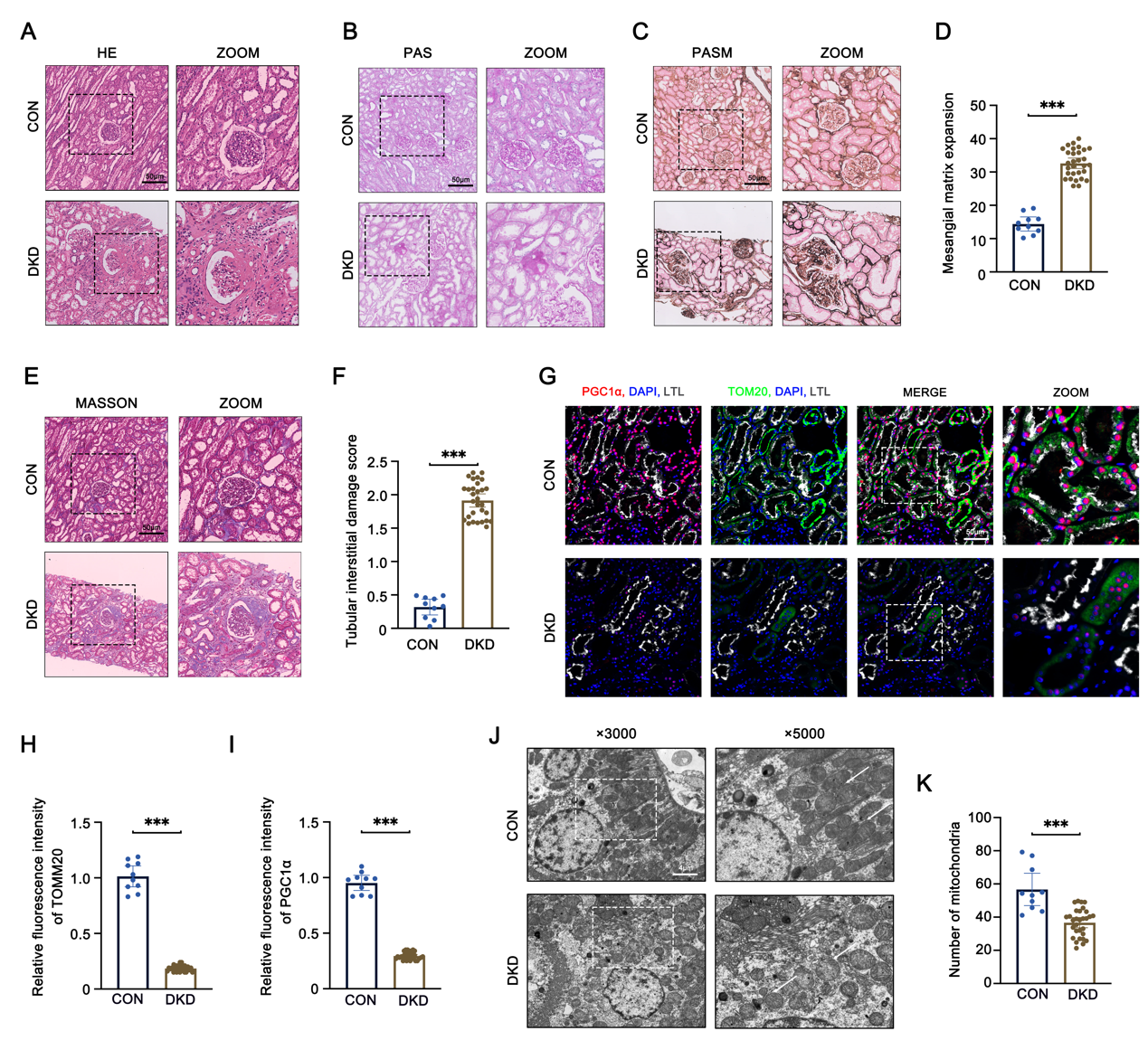
**

**Figure S1. Renal pathological changes in DKD patients. A)** Representative microscopic images of HE staining of kidney sections in DKD (n=30) and control (n=10) group patients. **B)** Representative microscopic images of PAS staining of kidney sections in DKD (n=30) and control (n=10) group patients. **C)** Representative microscopic images of PASM staining of kidney sections in DKD (n=30) and control (n=10) group patients. **D)** Mesangial matrix expansion of kidney sections in DKD (n=30) and control (n=10) group patients. **E)** Representative microscopic images of Masson staining of kidney sections in DKD (n=30) and control (n=10) group patients. **F)** Tubular interstitial damage score of kidney sections in DKD (n=30) and control (n=10) group patients. **G)** Representative immunofluorescent images and quantification of LTL (gray) and ERRα (red) in PTCs from DKD (n=30) and control (n=10) group patients. **H)** Relative fluorescence intensity of TOMM20 of kidney sections in DKD (n=30) and control (n=10) group patients. **I)** Relative fluorescence intensity of PGC1α of kidney sections in DKD (n=30) and control (n=10) group patients. **J-K)** TEM analyses of PTCs mitochondria in DKD (n=30) and control (n=10) group patients. The number and area of mitochondria per unit (per ×5000 field of view for mitochondria number). Data are presented as mean ± SEM. ****P* < 0.001.


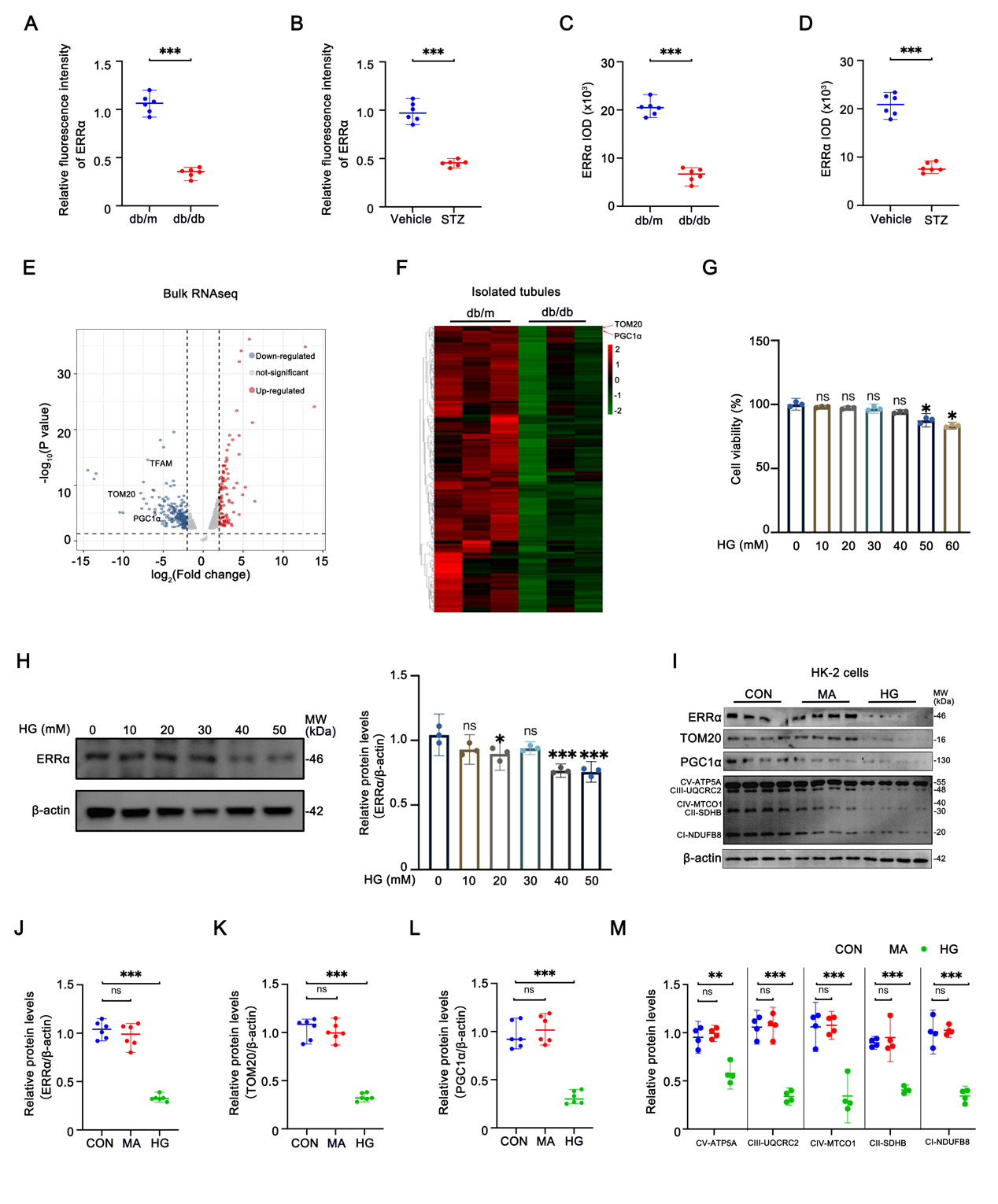


**Figure S2. ERRA expression is downregulated in DKD. A)** Quantification of LTL (gray) and ERRα (red) in PTCs from db/m (n=6) and db/db (n=6) mice. **B)** Quantification of LTL (gray) and ERRα (red) in PTCs from vehicle (n=6) and STZ (n=6) mice. **C)** Immunohistochemical semi-quantitative IOD analysis of ERRα in db/m (n=6) and db/db (n=6) mice. **D)** Immunohistochemical semi-quantitative IOD analysis of ERRα in vehicle (n=6) and STZ (n=6) mice. **E)** Volcano plot of RNA-seq in renal tubules of db/m and db/db mice. Red represents the upregulated genes and green represents the downregulated genes. **F)** Mitochondrial-related genes expression profiles were compared between renal tubules of db/m and db/db mice. **G)** Cell viability of different group of HK-2 Cells (n=3). **H)** Representative Western blot and densitometric analysis of ERRα after treating with high glucose stimulation at different concentrations for 24 hours. **I-M)** Representative Western blot and densitometric analysis of ERRα, TOM20, PGC1α and OXPHOS (CI-NDUFB8, CII-SDHB, CIII-UQCRC2, CIV-MTCO1 , CV-ATP5A) in different group of HK-2 cells (n=3). Data are presented as mean ± SEM. ns: *P* >0.05; **P* <0.05; ** *P* <0.01; *** *P* < 0.001.


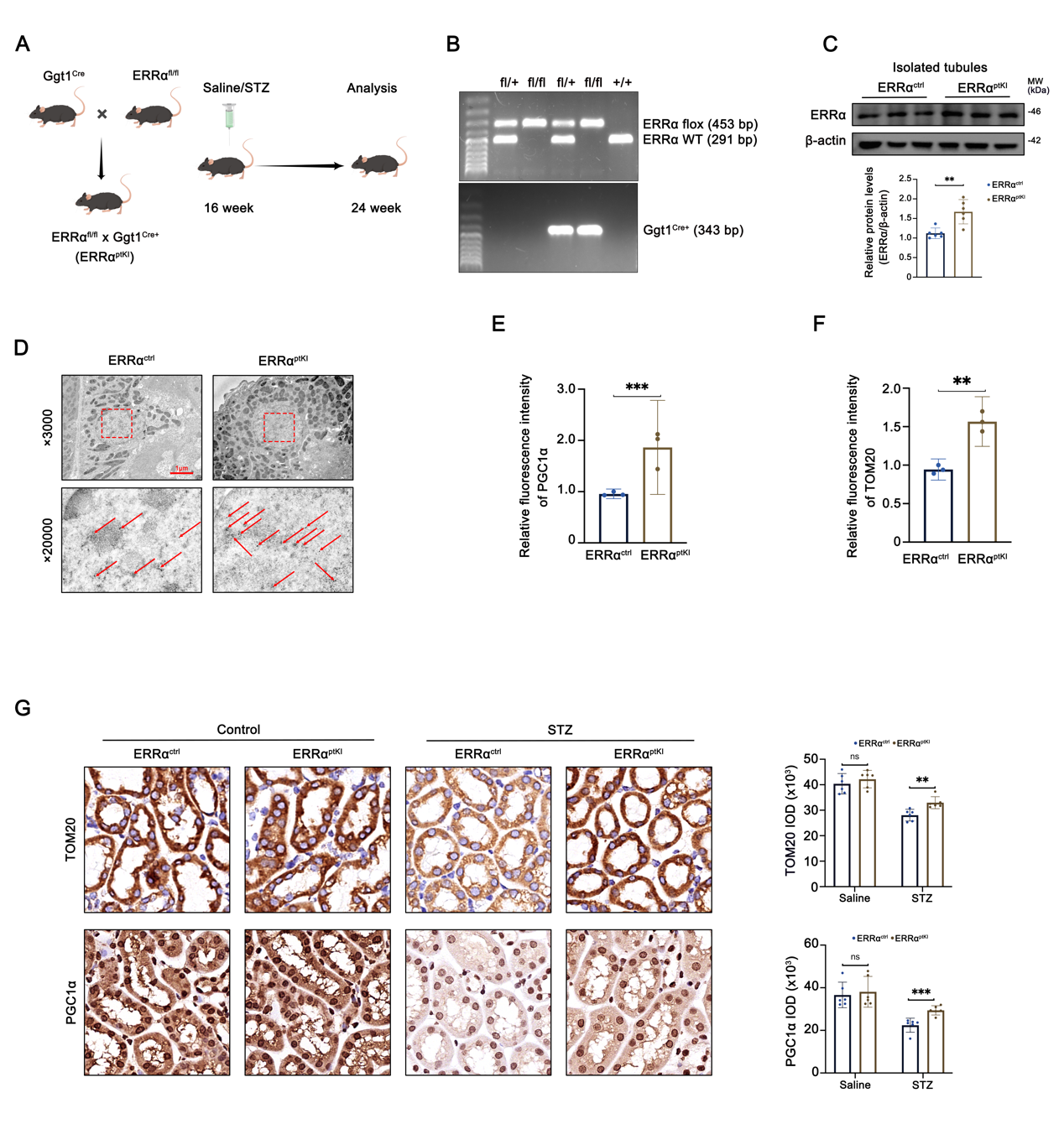


**Figure S3. Construction of ERRα^ptKI^ mice** **A)** A schematic diagram showing the construction of conditional knock-in of ERRα in PTCs of mice (ERRα^ptKI^ mice). **B)** PCR with genomic DNA from tail tissues as templates for verification of the floxed mouse. **C)** Representative Western blot and densitometric analysis of expression of ERRα in renal tubules from mice (n=6). **D)** TEM of immuno-gold labeling of ERRα of PTCs ultrastructure in ERRα^ptKI^ (n=6) and ERRα^ctrl^ mice**. E-F)** Relative fluorescence intensity of TOMM20 and PGC1α in ERRα^ctrl^ and ERRα^ptKI^ primary PTCs. **G)** Representative immunohistochemistry images and quantification of TOM20 and PGC1α in PTCs from different group of mice (n=6). Data are presented as mean ± SEM. ns: *P* >0.05; ***P* <0.01; ****P* < 0.001.


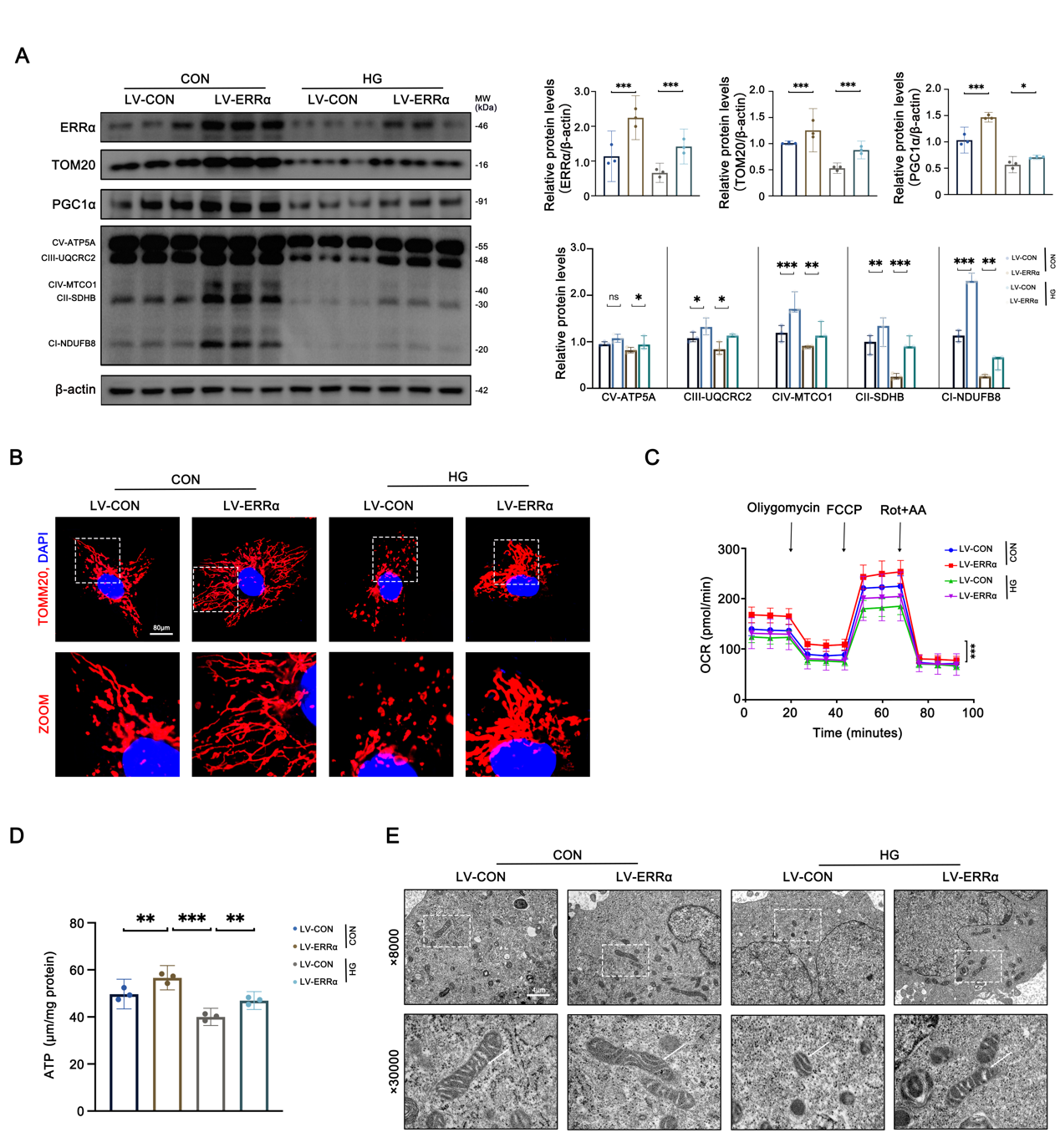


**Figure S4. Overexpression of ERRα attenuates HG-stimulated mitochondrial damage. A)** Representative Western blot and densitometric analysis of expression of ERRα, TOM20, PGC1α and OXPHOS in different group of HK-2 cells (n=3). **B)**. Representative immunofluorescent images of TOM20 (red) in in different group of HK-2 cells (n=3). **C)** Measurement of oxygen consumption rate (OCR) in different group of HK-2 cells (n=3). **D)** ATP content of different groups of HK-2 cells (n=3). **E)** TEM in different group of HK-2 cells (n=3). Data are presented as mean ± SEM. ns: *P* >0.05; **P* <0.05; ** *P* <0.01; *** *P* < 0.001.


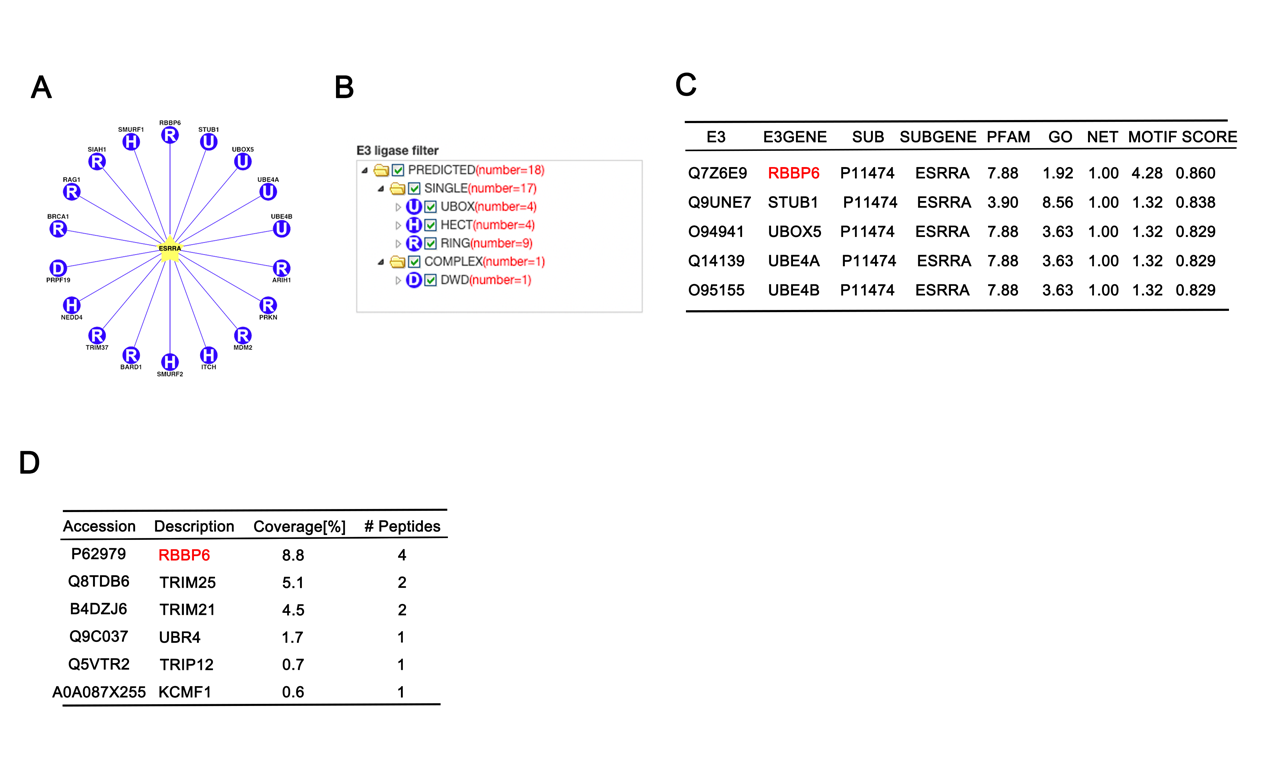


**Figure S5. Identification of the E3 ligase of ERRα. A-B)** A total of 19 E3 ligases were predicted by UbiBrowser by querying ERRα as substrate. **C)** Five E3 ligases with the highest confidence scores. **D)** RBBP6 was identified as the E3 ligase of ERRA by Mass spectrometry.


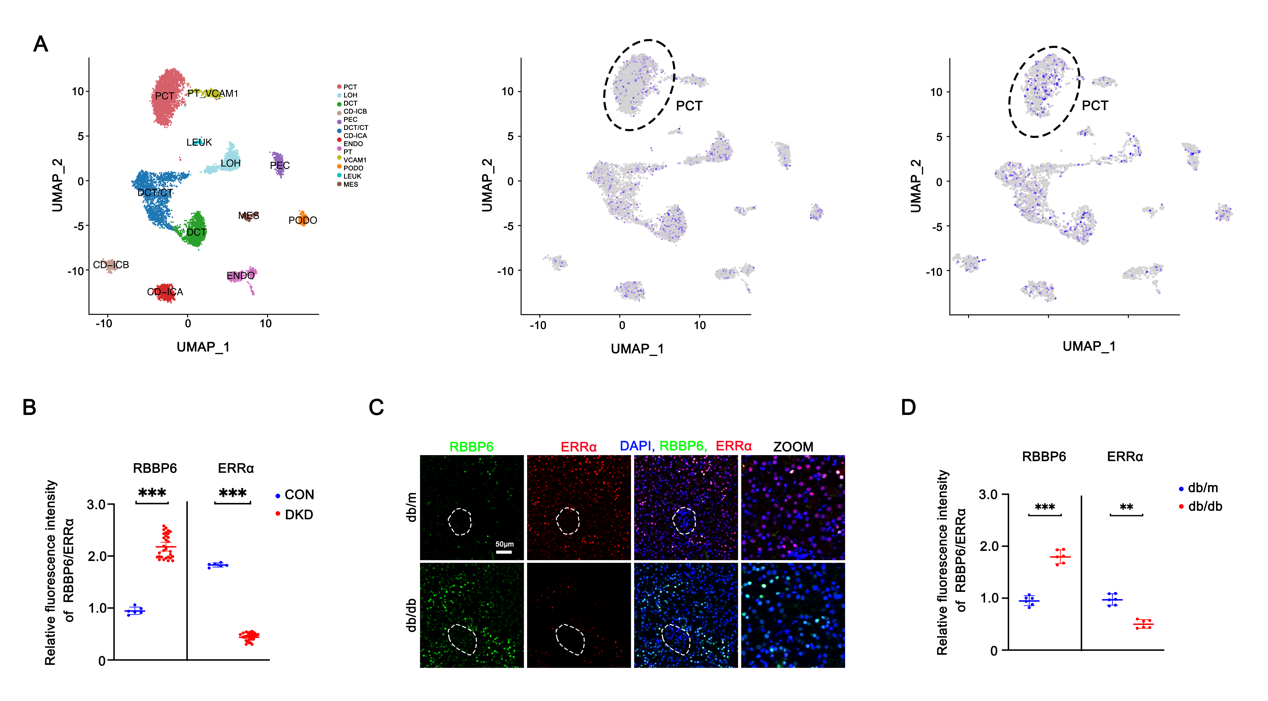


**Figure S6. Upregulated-RBBP6 and downregulated-ERRα was indicated in in animal models of DKD.** **A)** RBBP6 gene expression in healthy and DKD patients from single-nucleus RNA-seq. RBBP6 Expression Comparison across Clusters in healthy and DKD group. (GEO ID: GSE181382) **B)** Relative fluorescence intensity of RBBP6 and ERRα in DKD (n=30) and control (n=10) group patients. **C)** Representative immunofluorescent images of RBBP6 (green) and ERRα (red) of PTCs in db/m (n=6) and db/db (n=6) mice. Scale bars: 10 μm. The white dotted circles represent glomeruli. **D)** Relative fluorescence intensity of RBBP6 and ERRα in db/m (n=6) and db/db (n=6) mice. Data are presented as mean ± SEM.** *P* < 0.01; *** *P* < 0.001.


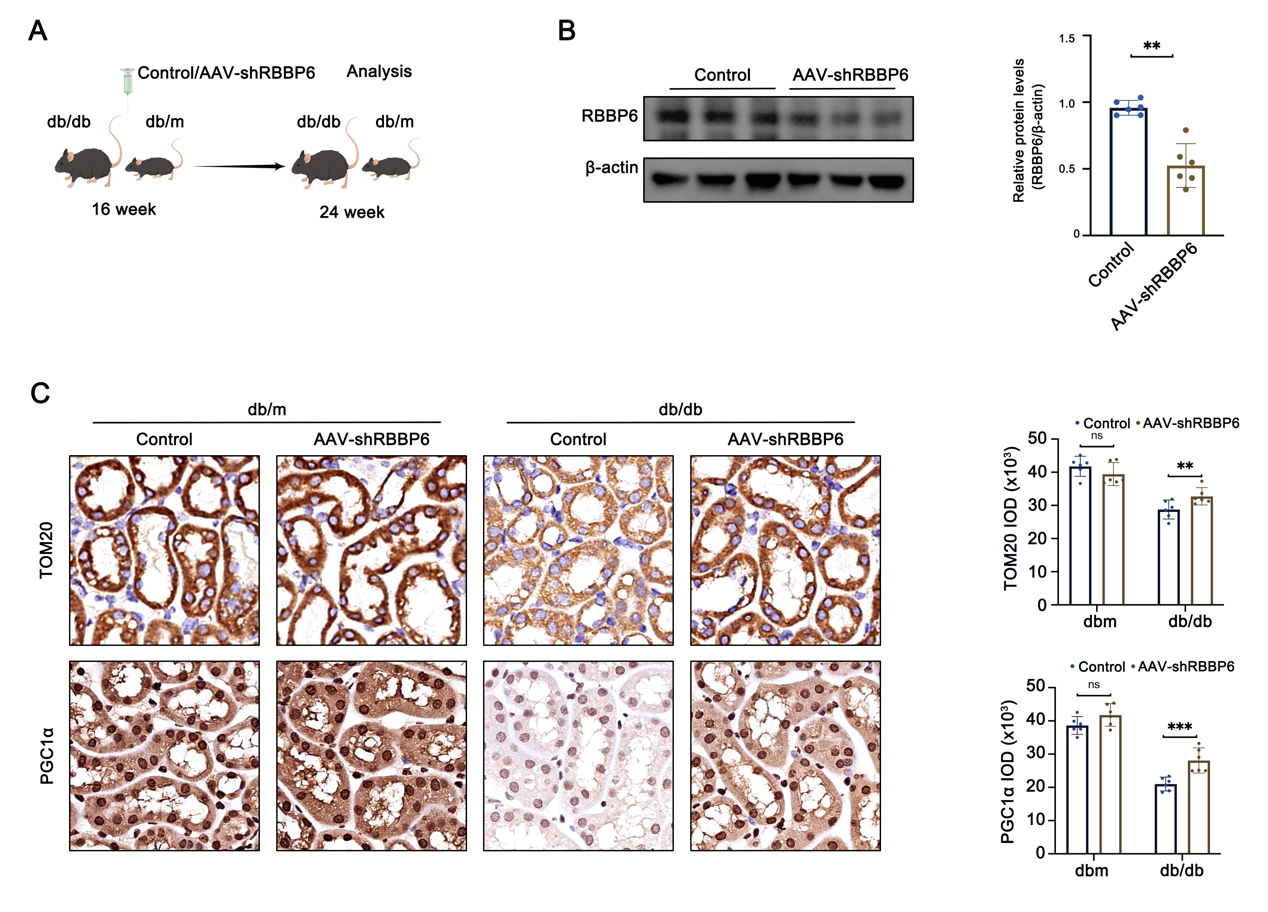


**Figure S7. Quantification of relative protein expression in different groups of mice after RBBP6 knockdown.** **A)** Schematic of the experiment. db/m or db/db mice were injected with control or AAV-shRBBP6 *via* in situ injection. **B)** Representative Western blot and densitometric analysis of RBBP6 expression in renal tubules from mice (n=6). **C)** Representative immunohistochemistry images and quantification of TOM20 and PGC1α in PTCs from different group of mice (n=6). Data are presented as mean ± SEM. ns: *P* >0.05; ** *P* <0.01; *** *P* < 0.001.


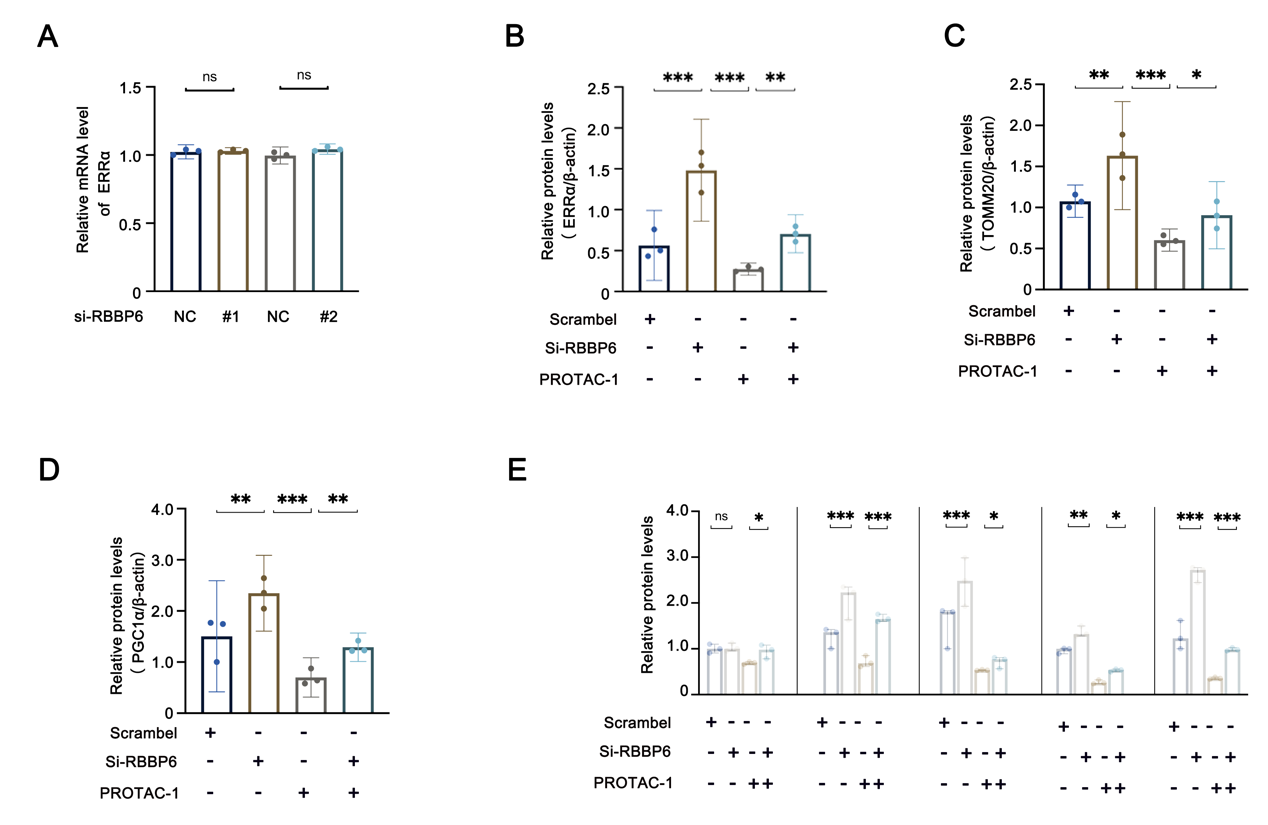


**Figure S8. Quantification of relative protein expression in different groups of HK-2 cells.** **A)** Relative mRNA levels of ERRαin different groups of HK-2 cells (n=3). **B-E)** Densitometric analysis of expression of ERRα, TOM20, PGC1α and OXPHOS (CI-NDUFB8, CII-SDHB, CIII-UQCRC2, CIV-MTCO1 , CV-ATP5A) in different groups of HK-2 cells (n=3). Data are presented as mean ± SEM. ns: *P* >0.05; **P* <0.05; ** *P* <0.01; *** *P* < 0.001.


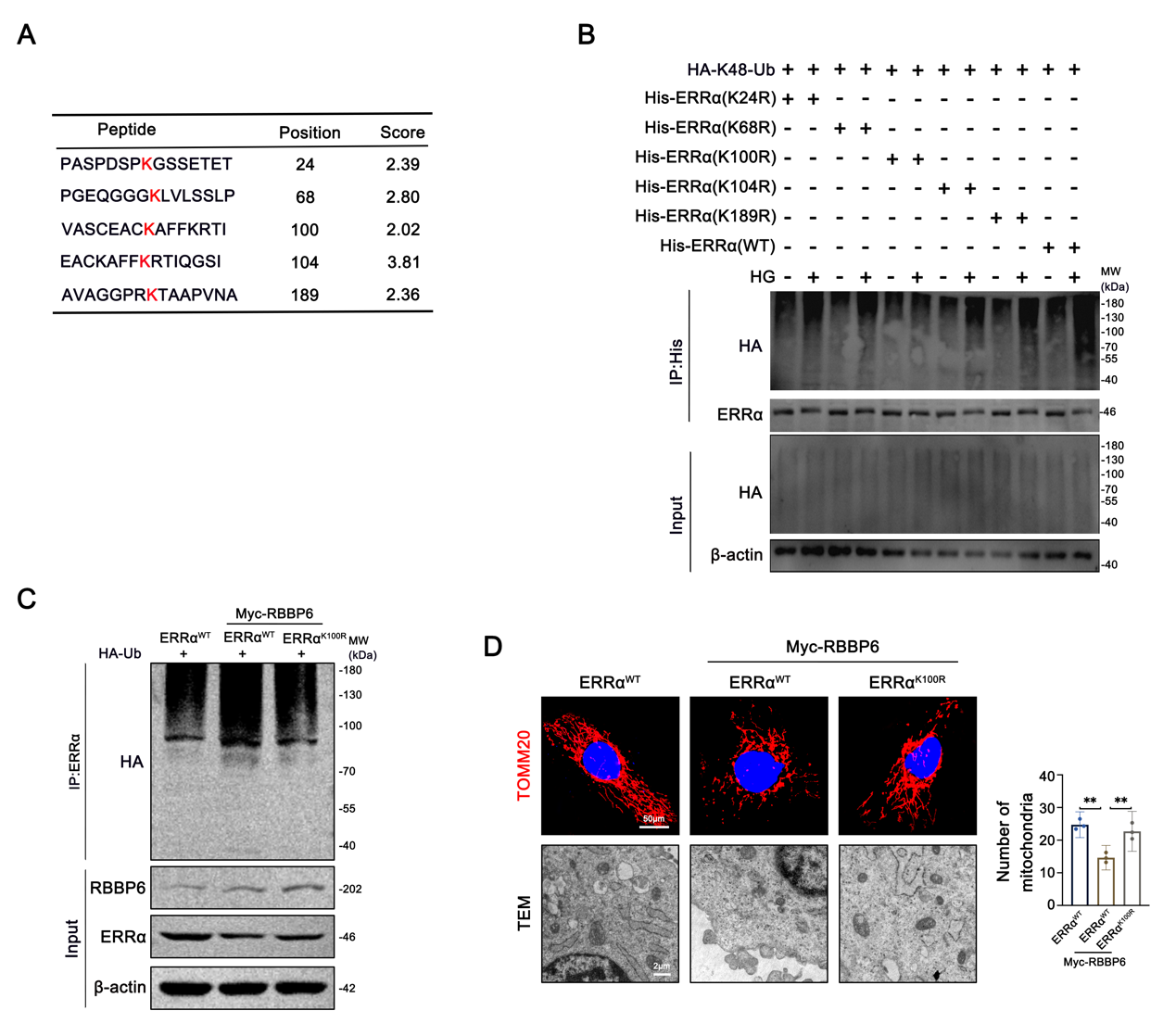


**Figure S9. The K100 residue is important for ubiquitination of ERRα.** **A)** Five possible ubiquitination residues with the highest confidence were selected for further validation. **B)** Representative Western blot of expression of ERRα in in different groups of HK-2 cells (n=3). **C)** Representative Western blot of expression of HA, RBBP6 and ERRα in different groups of PTCs (n=3). **D)** Representative immunofluorescent images of TOM20 (red) in different groups of PTCs (n=3). Data are presented as mean ± SEM. ** *P* <0.01.


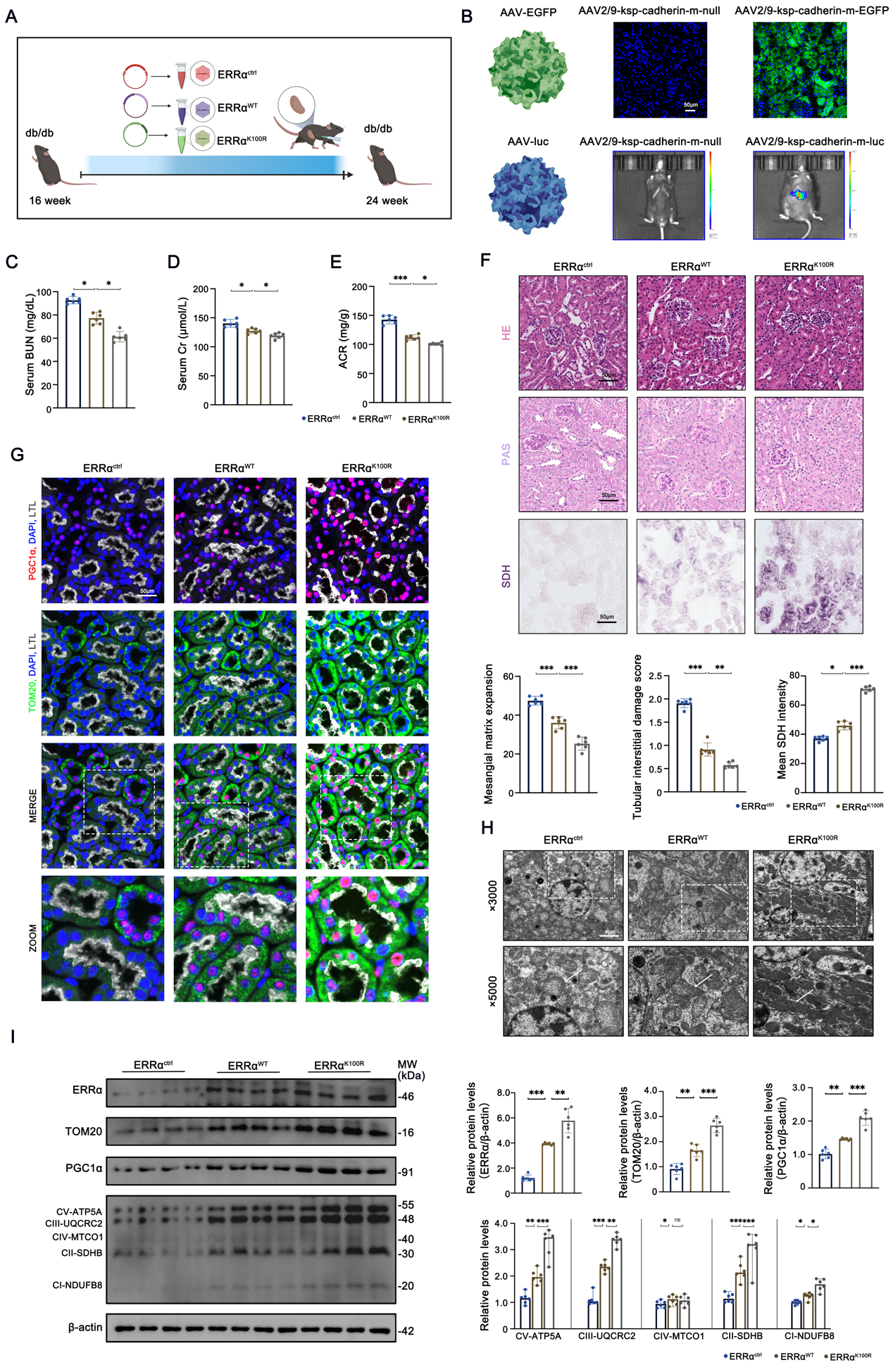


**Figure S10. The K100 residue is important for ubiquitination of ERRα.** **A)** Pattern diagram of AAV in situ injection **B)** Fluorescent staining and imaging of mice after AAV injection. **C-E)** BUN, SCr and ACR levels in different groups of mice (n=6). **F)** Representative images of HE, PAS, Masson and SDH stainings from different group of mice (n=6). **G)** Representative immunofluorescent images of LTL (gray), PGC1α (red) and TOM20 (green) in PTCs from different group of mice (n=6). **H)** TEM of PTCs ultrastructure in different group of mice (n=6). **I)** Representative Western blot and densitometric analysis of ERRα, TOM20, PGC1α, and OXPHOS (CI-NDUFB8, CII-SDHB, CIII-UQCRC2, CIV-MTCO1 , CV-ATP5A) in renal tubules from different group mice (n=6). Data are presented as mean ± SEM. ns: *P*>0.05; **P* < 0.05; ***P* < 0.01; ****P* < 0.001.

**Supplementary tables**

**Table S1. Clinical information from patients with DKD.**

| Patient | Age (year) | Gender(Male/female) | eGFR(ml/min/1.73m^2^) | ACR(mg/g) | ALB (g/L) |
| --- | --- | --- | --- | --- | --- |
| 1 | 46 | F | 11 | 63 | 3 |
| 2 | 61 | M | 13 | 24 | 10 |
| 3 | 66 | M | 25 | 54 | 22 |
| 4 | 50 | M | 44 | 53 | 29 |
| 5 | 48 | F | 32 | 15 | 54 |
| 6 | 60 | M | 58 | 48 | 35 |
| 7 | 55 | M | 82 | 34 | 15 |
| 8 | 61 | F | 39 | 40 | 32 |
| 9 | 49 | F | 84 | 39 | 12 |
| 10 | 67 | M | 85 | 24 | 36 |
| 11 | 47 | M | 48 | 16 | 13 |
| 12 | 66 | F | 83 | 35 | 46 |
| 13 | 62 | F | 52 | 38 | 25 |
| 14 | 47 | F | 94 | 48 | 28 |
| 15 | 63 | M | 54 | 25 | 12 |
| 16 | 51 | F | 68 | 58 | 67 |
| 17 | 51 | F | 49 | 28 | 78 |
| 18 | 61 | M | 72 | 25 | 38 |
| 19 | 60 | M | 58 | 46 | 55 |
| 20 | 57 | M | 67 | 13 | 57 |
| 21 | 55 | M | 47 | 36 | 54 |
| 22 | 60 | M | 49 | 12 | 65 |
| 23 | 64 | F | 58 | 19 | 60 |
| 24 | 49 | M | 89 | 27 | 54 |
| 25 | 51 | F | 73 | 32 | 78 |
| 26 | 51 | F | 93 | 43 | 33 |
| 27 | 66 | M | 87 | 20 | 38 |
| 28 | 58 | M | 110 | 19 | 56 |
| 29 | 46 | F | 76 | 14 | 48 |
| 30 | 57 | M | 130 | 25 | 82 |

**Table S2. Primers used in Real-time PCR.**

| Gene | Species | Forward | Reverse |
| --- | --- | --- | --- |
| *ERRα* | mouse | ACTGCAGAGTGTGTGGATGG | ACGGAGTCAGAGTTGGCAAG |
| *β-actin* | mouse | GTAAAGACCTCTATGCCAACA | GGACTCATCGTACTCCTGCT |
